# NUDT7 Modulates the UBA52-SREBF1 Signaling Axis to Promote PRRSV Replication via Lipid Synthesis

**DOI:** 10.7150/ijbs.127844

**Published:** 2026-01-14

**Authors:** Yuchao Yan, Changyan Li, Junyang Zhang, Ruiqiao Li, Boxuan Yin, Sicheng Zhang, Yinhao Zhang, Xinyi Zhang, Jun Han, Jinhai Huang

**Affiliations:** 1 School of Life Sciences, Faculty of Medicine, Tianjin University, Tianjin, 300072, China.; 2 Laboratory Animal Center, Tianjin University of Traditional Chinese Medicine, Tianjin, 301617, China.; 3 National Key Laboratory of Veterinary Public Health Safety, College of Veterinary Medicine, China Agricultural University, Beijing, China.

**Keywords:** PRRSV, NUDT7, lipid metabolism, ubiquitin, innate immunity

## Abstract

Identifying cellular proteins and processes crucial for viral infection is vital for comprehending virus-induced disease mechanisms and developing host-targeted therapies. PRRSV has been shown to take advantage of host metabolic reprogramming and immunosuppression to promote virus production, but the host factors that coordinate these processes have not been fully elucidated. Here, we showed that NUDT7 expression was significantly increased during PRRSV infection by ETS1 targeting its promoter. We also found NUDT7 enhance PRRSV replication by reprograms viral infection-induced intracellular lipid droplets (LDs) synthesis. Mechanistically, NUDT7 interacts with and targets the ubiquitin-ribosomal fusion protein UBA52 for proteasomal degradation. NUDT7 enhances lipid droplet formation and stabilizes the lipogenic transcription factor SREBF1 by blocking UBA52-mediated K11/K27/K48 polyubiquitination. NUDT7-UBA52-SREBF1 axis drives lipid metabolic reprogramming, creating a favorable environment for PRRSV replication. Additionally, NUDT7 inhibits type I interferon signaling and the expression of interferon-stimulated genes, facilitating viral immune evasion. These findings suggest that NUDT7 could be a therapeutic target for combating PRRSV infection, offering a novel perspective and theoretical foundation for developing targeted metabolic-immune antiviral strategies.

## Introduction

PRRSV is a single-stranded RNA virus from the Arteriviridae family. Porcine reproductive and respiratory syndrome (PRRS), a major viral disease impacting swine globally, is primarily caused by the PRRS virus (PRRSV). PRRSV predominantly manifests as reproductive failure in breeding sows, leading to decreased litter sizes, increased stillbirths, and delayed estrus. Additionally, PRRSV induces respiratory issues in piglets, characterized by coughing, dyspnea and increased susceptibility to secondary bacterial infections^1^,^2^. PRRSV is a single-stranded RNA virus with a genome approximately 15.4 kilobases long. The genome contains at least 11 unique open reading frames (ORFs) responsible for producing 16 non-structural and 8 structural proteins, each crucial to the virus's life cycle and pathogenicity^3^,^4^. As an immunosuppressive pathogen, PRRSV encodes proteins that markedly weaken the host's innate immune response through various strategies^5^. Non-structural protein 2 (Nsp2) among the viral proteins significantly counteracts the host's innate immune response via two primary molecular mechanisms. It acts as a deubiquitinase, removing ubiquitin from key immune signaling molecules like RIG-I and IκBα, while also detaching ISG15 from its substrates and reducing cellular ISG15 protein levels. Furthermore, the frameshift-derived products Nsp2TF and Nsp2N disrupt the assembly of the mitochondrial antiviral signaling protein (MAVS) complex and hinder the formation of the interferon-stimulated gene factor 3 (ISGF3) complex, weakening host antiviral defenses.^6^-^8^. Effective vaccines and antiviral drugs for this virus are currently unavailable. Consequently, further investigation into the interaction mechanisms between PRRSV and hosts is crucial for developing effective preventive and therapeutic strategies.

Recent evidence highlights the crucial role of reprogrammed intracellular metabolism in regulating host defense activation following viral invasion. Lipid metabolism is integral to viral budding, invasion, replication, and is closely linked to viral immune evasion^9^,^10^. The deletion of fatty acid synthesis genes Stearoyl-CoA Desaturase-2 (SCD2) and Acetyl-CoA Carboxylase (ACC) reduces monounsaturated fat (MUFA) production, activating the cGAS-STING pathway and enhancing interferon-stimulated gene (ISG) expression, which increases the antiviral activity against Influenza A virus (IAV)^11^,^12^. The SREBF family plays a crucial role in lipid metabolism, influencing both viral proliferation and the transcription of immune-related genes^13^,^14^. Lipid droplets, as the main organelles for lipid storage, act as molecular switches in anti-infection immune response and regulates immune cell metabolism. Research indicates that lipid droplets function as both an assembly platform and a source of lipids and energy essential for viral replication. Coronavirus ORF6 promotes fatty acids into mitochondrial β-oxidation to produce ATP by enhancing lipid droplet synthesis and degradation, providing energy for SARS-CoV-2 replication^15^. O-GlcNAc transferase (OGT) integrates innate immunity and lipid metabolism to inhibit viral replication by binding the IAV genome to promote K48 ubiquitination degradation of lipid droplet Perilipin 2 (PLIN2)^16^. Lipid metabolism plays a role in the PRRSV life cycle and is linked to cellular inflammation and immune responses. Fatty acids influence the palmitoylation of GP5 and M proteins, impacting PRRSV assembly and release^17^,^18^. PRRSV Nsp4 modulates cholesterol metabolism in cells and suppresses IFN-I production^19^,^20^. PRRSV infection suppresses NDRG1 expression and triggers lipophagy, facilitating viral replication and assembly. Inhibiting lipid droplets significantly reduces PRRSV replication, NF-κB signaling, and down-regulates IL-1β and IL-8 transcription^21^,^22^. Investigating the interplay between lipid metabolism and PRRSV enhances comprehension of viral replication mechanisms and offers novel insights for anti-PRRSV drug development.

Nudix hydrolase, a class of enzymes predominantly localized in peroxisomes, exerts dual biological functions: on one hand, it scavenges cytotoxic nucleotide metabolites to maintain intracellular metabolic homeostasis; on the other hand, it precisely modulates the intracellular concentrations of a diverse array of nucleotide-derived substrates, metabolic cofactors, and signaling molecules^23^. NUDT7, a member of the nudix hydrolase family, functions as a CoA diphosphatase, facilitating CoA cleavage. Deletion of NUDT7 disrupts lipid homeostasis in chondrocytes and increases inflammatory factor expression, resulting in chondrocyte death^24^. In fasting murine models, ectopic NUDT7 expression reduces peroxisomal fatty acid β-oxidation rates, suggesting its regulatory role in hepatic peroxisomal lipid metabolism^25^,^26^. Beyond its metabolic functions, NUDT7 is also implicated in the orchestration of inflammatory responses; notably, the genetic ablation of NUDT7 can induce marked alterations in the cellular antioxidant status, thereby highlighting its pleiotropic roles in maintaining cellular homeostasis^27^. Here, we report that PRRSV infection enhances NUDT7 expression and promotes viral replication. NUDT7 modulates intracellular lipid droplet synthesis by degrading UBA52 to influence SREBF1 expression, while also suppressing the IFN-I immune response and facilitating PRRSV replication. Our research is of great significance for further understanding the interaction between pathogen infection and host metabolic mechanisms, and inspires new antiviral strategies.

## Materials and Methods

### Cells and viruses

HEK293T (ATCC, CRL-11268) and Marc-145 (ATCC, CRL-12231) cells were maintained in DMEM (Gibco, C11995500BT) with 10% FBS (PAN-Biotech, P30-1406). iPAMs, an immortalized porcine alveolar macrophage cell line expressing the PRRSV receptor CD163, were cultured in RPMI 1640 medium (Gibco, catalog no.).C22400500BT) with 10% fetal bovine serum (FBS) added. Primary porcine alveolar macrophages (PAMs) were extracted from 30-day-old pathogen-free healthy pigs and cultured using RPMI 1640 medium (Gibco, catalog no.C22400500BT), supplemented with 10% FBS and 1% penicillin-streptomycin to prevent bacterial contamination. Cell cultures were maintained at 37 °C in a humidified environment with 5% CO₂. The viral strains used in this study—PRRSV strain JXwn06, vesicular stomatitis virus (VSV), porcine circovirus type 2 (PCV2), and porcine epidemic diarrhea virus (PEDV)—were preserved in our laboratory.

### Antibodies and reagents

Antibodies including anti-SREBF1, anti-ACC1, anti-FASN, anti-PPARγ, anti-ATGL, anti-PGC1A, anti-Actin, anti-MDA5, anti-Flag, anti-mouse IgG-HRP-linked, anti-rabbit IgG-HRP-linked, and anti-GAPDH were sourced from Proteintech. Antibodies including anti-ETS1 (14069), anti-RIG-I (3743), anti-p-TBK1 (5483), anti-TBK1 (3504), anti-P65 (8242), anti-p-P65 (3033), anti-Ubiquitin (58395), and anti-HA (3724) were sourced from Cell Signaling Technology. Anti-NUDT7 (LS-C473751) was purchased from Lsbio. Anti-Catalase (PB0971), anti-PMP70 (BA3339) were obtained from Boster. Anti-Myc (AE070), anti-Flag (AE063) and anti-UBA52 (A20876) were obtained from ABclonal. The anti-PRRSV nsp2 antibody was provided by Prof. Han Jun from China Agricultural University. Anti-PRRSV N protein pAb and NUDT7 pAb were developed and preserved in our laboratory.

The compounds MG132 (HY-13259), Chloroquine (HY-17589A), BODIPY 493/503 (HY-W090090), Fatostatin (HY-14452), and Bafilomycin A1 (HY-100558) were sourced from MedChemExpress. Oil Red O (O0625) and Hoechst33258 (94403) were sourced from Sigma-Aldrich.3-Amino-1,2,4-triazole (S5977) was obtained from Selleck. Cell culture dish (CCD06-060A), Cell culture plate (CCP06-006), Sterile centrifuge tube (ATS05-50) were purchased from Bioland.

### Plasmid construction and RT-qPCR

All eukaryotic expression vectors utilized in this study were engineered via the seamless cloning technique. The detailed operational protocols for vector construction were consistent with the standardized procedures previously established and validated in our research laboratory. For RNA extraction, cells cultured under distinct experimental conditions were harvested and lysed with TRIzol reagent (TaKaRa, Cat. No. 9109) following the standard protocol for total RNA isolation. Subsequently, a sequential addition of chloroform, isopropanol, and DEPC-treated anhydrous ethanol was performed to separate, precipitate, and purify the RNA fraction, respectively. After each treatment step, the samples were subjected to high-speed centrifugation at 12,000 rpm for 15 minutes to facilitate phase separation or nucleic acid pellet formation. Following RNA purification, complementary DNA (cDNA) was synthesized through reverse transcription using a commercial reverse transcription kit (Japan Biotechnology Corporation, Cat. No. 11123ES60), with all operations strictly adhering to the manufacturer's recommended protocol. Quantitative real-time polymerase chain reaction (RT-qPCR) was then conducted to quantify target gene expression levels; the reaction system was prepared in sterile PCR tubes (Bioland, Cat. No. PCR001-8F) using the Hieff®qPCR kit (Yesen Biotechnology, Cat. No. 11202ES08). The thermal cycling program was set as follows: an initial denaturation step at 95 °C for 5 min, followed by 45 cycles of denaturation at 95 °C for 10 sec, annealing at 57 °C for 20 sec, and extension at 72 °C for 34 sec. The nucleotide sequences of the gene-specific primers employed in this RT-qPCR assay are listed in Table S1.

### Immunoblot analysis

Briefly, total cellular proteins were extracted from harvested cell samples via lysis with radioimmunoprecipitation assay (RIPA) buffer (Solarbio, Cat. No. R0100), which was supplemented with a protease inhibitor cocktail to prevent protein degradation during the extraction process. After lysis, the protein concentration of each sample was accurately quantified using a bicinchoninic acid (BCA) protein assay kit (Solarbio, Cat. No. PC0020), with bovine serum albumin (BSA) serving as the standard reference for calibration curves.

Denatured protein lysates were equally loaded onto SDS-PAGE gels of varying concentrations, chosen according to the target proteins' molecular weights to optimize separation efficiency. After electrophoresis, proteins were transferred to polyvinylidene fluoride (PVDF) membranes using a wet transfer method at a constant voltage for 90 minutes. Membranes were incubated in a 5% non-fat skim milk blocking buffer at room temperature for 1 hour with gentle shaking to prevent non-specific binding.

The blocked PVDF membranes were incubated overnight at 4 °C with primary antibodies diluted in blocking buffer at optimal concentrations established in preliminary experiments. The membranes underwent three washes with Tris-buffered saline with Tween-20 (TBST) before being incubated with horseradish peroxidase (HRP)-conjugated secondary antibodies at room temperature for 1 hour. Specific target protein bands were ultimately visualized using an enhanced chemiluminescence (ECL) detection reagent. Chemiluminescent signals were captured using a gel imaging system for densitometric analysis.

### Indirect immunofluorescence assay (IFA)

Cells were inoculated in a 6-well plate with a climbing plate and subjected to various experimental treatments before being harvested. Cells were fixed with 4% paraformaldehyde (Solarbio, P1110) at room temperature for 30 minutes, followed by three PBS washes. Cells were permeabilized using 0.5% Triton X-100 for 15 minutes, followed by blocking with 10% bovine serum albumin for 2 hours, and incubated overnight at 4 °C with the specific primary antibody. After washing with PBS for 3 times, the cells were incubated with fluorescently labeled secondary antibodies at 37 °C for 1 h. Finally, the nucleus was stained with Hoechst33258 for 15 min, and then an anti-quenching sealant was added dropwise to the slide. The slide was inverted, dried at room temperature in the dark, and stored at -20 °C. Fluorescence images were captured using a Leica SP8 confocal microscope and analyzed quantitatively with Image J software.

### Luciferase reporter assay

For immunofluorescence staining assay, cells were first seeded into 6-well plates. At 70-80% confluence, cells were co-transfected with the dual-luciferase reporter plasmid, pRL-TK (as an internal reference for normalizing transfection efficiency), and either an empty vector or a specific target plasmid using Lipofectamine 3000 (Invitrogen) following the manufacturer's protocol. Following the experimental treatments, cells were collected, and reporter gene activity was assessed using a dual-specific luciferase assay kit (Yeasen, Cat. No. 11402ES60).

### Co-IP

Cells were seeded in appropriate culture vessels and subjected to transient transfection with the specified recombinant plasmids according to the experimental design. Following a 24-hour transfection incubation, adherent cells were gently rinsed with pre-chilled phosphate-buffered saline (PBS) and lysed on ice for 30 minutes using immunoprecipitation (IP) lysis buffer. After lysis, cell lysates were centrifuged at 12,000 × g for 15 minutes at 4 °C to remove insoluble debris. For the immunoprecipitation procedure, the remaining supernatant fractions were incubated with the target-specific primary antibody or non-immune control immunoglobulin G (IgG) at 4 °C with gentle end-over-end rotation overnight. Magnetic beads, either Protein A/G-coupled or Flag-tagged based on the target protein's epitope tag, were added to the antibody-supernatant mixtures and incubated for an additional 4 hours at 4 °C to form stable antibody-antigen-bead complexes. Following the binding reaction, magnetic beads were isolated using a magnetic separation rack and washed five times with PBST buffer (PBS with 0.05% Tween-20) to remove non-specifically bound proteins. The washed beads were combined with 2× SDS protein loading buffer, then vortexed vigorously and heated at 95-100 °C for 10 minutes to elute and denature the immunoprecipitated proteins. The prepared samples, comprising both input and immunoprecipitated fractions, underwent SDS-PAGE and subsequent Western blot analysis to identify target proteins.

### CRISPR/Cas9 knockout and RNAi

The target sequence's double-stranded oligonucleotide was cloned into the lentii-crispr-v2/pLKO.1 vector and co-transfected with packaging plasmids pMD2.G and psPAX2 into HEK293T cells for 48 hours. Lentiviruses were collected at 24 and 48 hours. Cells were infected with lentiviruses and screened for 7 days using puromycin or Hygromycin B (4 μg/mL; Solarbio, P8230). Knockout efficiency was assessed via Western blotting. Chemically synthesized siRNA for RNA interference (RNAi) experiments was produced by Suzhou Jima Gene Co., Ltd. The indicator gene siRNA was transfected into cells for endogenous gene knockdown by using Lipofectamine 3000 (Invitrogen). Table S2 describes the information of primer sequences.

### Intracellular TG and TC assays

Cells were harvested through trypsin digestion and centrifugation. A 0.1 ml aliquot of lysate was added per 1x10^6 cells, thoroughly mixed, and incubated at room temperature for 10 minutes to achieve lysis. Transfer the supernatant to a 1.5 ml centrifuge tube, heat at 70 ºC for 10 minutes to induce floc precipitation, then centrifuge at 2000 rpm for 5 minutes at room temperature. These values were normalized to total cellular protein content, determined via the BCA protein assay kit (Beyotime, P0012). TG and TC levels were assessed using Applygen's triglycerase (E1013) and total cholesterolase (E1015) assay kits, following the provided protocols.

### LDs staining

Cells underwent Oil Red O staining by being washed thrice with PBS and fixed in 4% PFA for 30 minutes. The cells were then washed with PBS and permeabilized with 60% isopropyl alcohol for 15 min (rinsing to facilitate oil red O staining).A 60% diluted oil red O dye solution was prepared by mixing a 3:2 diluted saturated oil red O solution in isopropanol-water with ddH2O. It was then mixed, filtered, and left at room temperature for 10 minutes, resulting in a wine red color without any precipitation. Isopropanol was discarded, and the sample was air-dried for 2 minutes before being stained with oil red O for 30 minutes. Rinse 3 times with double distilled water, counterstain with hematoxylin, drop 15 ul of melted glycerin gelatin onto the slide, seal the slide.

In the BODIPY lipid staining assay, adherent cells on sterile coverslips were gently rinsed three times with ice-cold phosphate-buffered saline (PBS) to eliminate residual culture medium and non-adherent debris. Cells were fixed with 4% paraformaldehyde at room temperature for 30 minutes to preserve morphology and immobilize intracellular lipids. Following fixation, the PFA solution was discarded, and the cells were washed three times with PBS for 5 minutes each to remove any remaining fixative that might quench fluorescence signals or cause non-specific background staining. Next, the cells were incubated with BODIPY 493/503 dye (working concentration: 2 μg/mL, dissolved in PBS containing 0.1% Triton X-100 to enhance membrane permeability) in a dark, humidified chamber for 1 hour at room temperature. After staining, excess dye was eliminated through three PBS washes (5 minutes each) under dim light to reduce fluorescent probe photobleaching. The stained cell coverslips were mounted on glass slides with an anti-fade medium to enhance fluorescence stability. Fluorescent digital images were captured using a Leica SP8 confocal microscope with a 488 nm argon laser for excitation and a 510-530 nm band-pass filter for emission collection. All images were captured with consistent laser power, gain, and exposure time to maintain quantitative accuracy. The relative fluorescence intensity of BODIPY-stained lipid droplets in each sample was further quantified using ImageJ software, with at least 10 randomly selected fields of view analyzed per experimental group to ensure statistical reliability.

### LC-MS/MS analysis

Marc-145 cells were transfected with Flag-NUDT7 and infected with PRRSV for 24 hours to identify potential NUDT7-binding proteins. The cells were then collected, and NUDT7 was immunoprecipitated using Flag magnetic beads at 4 °C. IP products were examined using liquid chromatography-tandem mass spectrometry (LC-MS/MS) with a Q Exactive HF mass spectrometer and a Dionex Ultimate 3000, both from Thermo Fisher Scientific, USA.Mass spectrometry data were processed with MaxQuant software (https://www.maxquant.org/). MS assays were conducted by Beijing Protein Innovation Co., Ltd (China).

### Flow cytometry analysis and immunofluorescence microscopy

HEK293T cells infected with GFP-VSV were analyzed by direct immunofluorescence microscopy or flow cytometry. In short, once the cells were 80% confluent, the serum-free DMEM medium was swapped out, and GFP-VSV at varying infection levels was introduced and incubated for 1 hour. The cells were then rinsed twice with PBS and grown in DMEM medium with 2% FBS. To perform immunofluorescence microscopy analysis, the GFP-VSV virus was analyzed using an inverted fluorescence microscope. The magnification is ×200. Cells for flow cytometry analysis were collected using cold 1×PBS and analyzed with a FACS Calibur (BD Biosciences) featuring a 488 nm argon laser and a 635 nm red diode laser. Data analysis was conducted using FlowJo software (Ashland, OR, USA).

### Statistical analysis

Results are presented as means ± SEM. Statistical significance was determined by Student's t test/ANOVA using Prism 10 (GraphPad Software). Figures denote significant differences as follows: *P ≤ 0.05, **P ≤ 0.01, ***P ≤ 0.001.

## Results

### NUDT7 was upregulated by PRRSV infection through transcription factor ETS1

**Figure 1:**
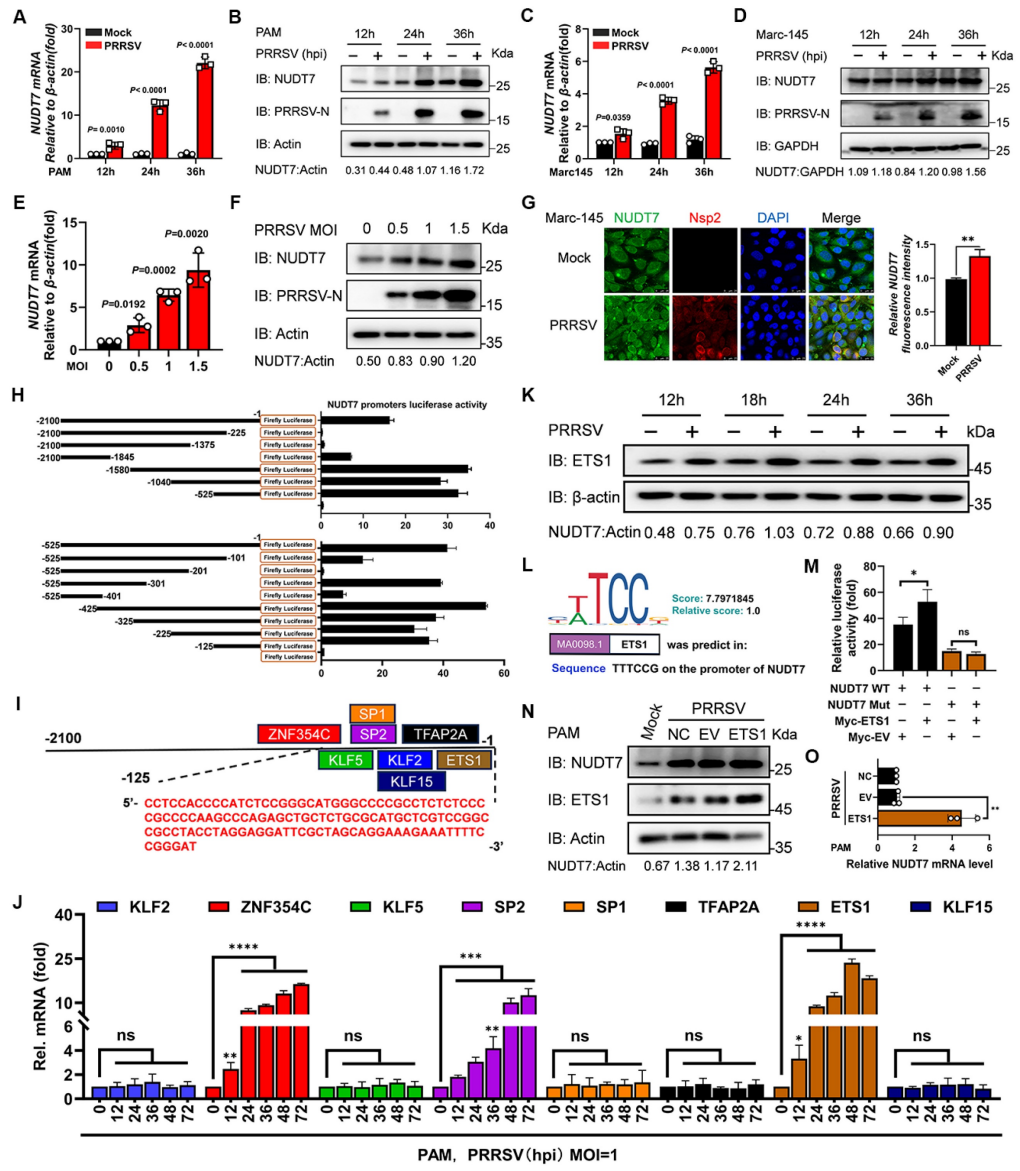
The transcription factor ETS1 regulates NUDT7 expression in PRRSV-infected cells. The expression of NUDT7 mRNA and protein in PAM cells (A-B) and Marc-145 cells (C-D) was assessed using RT-qPCR and Western blotting at intervals from 0 to 36 hours following PRRSV infection. (E-F) NUDT7 mRNA and protein levels in PAM cells were assessed 24 hours after PRRSV infection at MOIs of 0, 0.5, 1, and 1.5 using RT-qPCR and Western blotting. Western blot data were semi-quantified and normalized using β-actin as the loading control. (G) Immunohistochemical analysis of NUDT7 and PRRSV-Nsp2 expression in negative control and PRRSV-infected cells (left panel). Scale bar: 25 μm. The right panel presents quantitative data on the relative fluorescence intensity of NUDT7. (H) Analysis of luciferase activity in PAM cells after transfection with pGL3-Basic luciferase vector carrying a truncated NUDT7 promoter construct (-2100 to -1). (I) JASPAR-predicted transcription factor binding sites on the NUDT7 promoter. (J) Relative mRNA levels of predicted genes in PAM cells 24 hours post-infection (hpi) with PRRSV (assessed by RT-qPCR). (K) PAM cells infected with PRRSV at MOI=1 or mock-infected. Cells were harvested at specified time points. ETS1 expression was analyzed by Western blotting. ACTB served as the loading control. (L) JASPAR-generated ETS1 DNA motif sequence logo. (M) PAM cells co-transfected with designated reporter plasmids and phRL-TK; cells harvested after 48 hours for dual luciferase assay. (N-O) NUDT7 relative levels in the ETS1 overexpressed PAM cells explored using western blotting and RT-qPCR.

The function of NUDT7 had not been investigated in the field of the virus. To delve into the potential function of NUDT7 in PRRSV infection, we infected the main target cells in pigs, namely, porcine alveolar macrophages (PAMs), and then analyzed NUDT7 expression. We found that virus infection markedly increased the expression of NUDT7 in a time- (Fig. 1A-B) and dose- (Fig. 1E-F) dependent manner. Furthermore, to investigate whether NUDT7 was upregulated by PRRSV infection in other cell lines, we infected Marc-145 cells with PRRSV, and found that NUDT7 was also upregulated during PRRSV infection (Fig. 1C-D). Concomitantly, immunofluorescence assay (IFA) confirmed the similar results (Fig. 1G). These data demonstrate that NUDT7 expression is induced by PRRSV infection.

To elucidate the underlying molecular mechanism by which porcine reproductive and respiratory syndrome virus (PRRSV) mediates the upregulation of NUDT7 expression, we first focused on identifying the functional promoter region of the NUDT7 gene. A 2100-base pair (bp) full-length promoter fragment of NUDT7, along with a series of truncated promoter sequences, was amplified via polymerase chain reaction (PCR) using specific primers. These amplified fragments were then individually cloned into the pGL3-Basic luciferase reporter vector, which lacks a promoter and enhancer, to construct recombinant luciferase reporter plasmids. Subsequently, the recombinant plasmids were transfected into appropriate host cells together with the pRL-TK renilla luciferase plasmid (as an internal reference to normalize transfection efficiency). After 24 h of transfection, the dual-luciferase reporter assay system was employed to detect the relative luciferase activity of each group. The results demonstrated that among all the cloned NUDT7 promoter fragments, the segment spanning nucleotides -525 to -1 (relative to the transcription start site) exhibited significantly higher luciferase activity compared to other truncated fragments, indicating that this region contains key regulatory elements responsible for driving NUDT7 transcription (Fig. 1H). To further narrow down the range of the NUDT7 core promoter sequence, the -525 to -1 bp fragment was subjected to secondary truncation to generate a set of shorter overlapping fragments. These secondary truncated fragments were also cloned into the pGL3-Basic vector to construct new recombinant reporter plasmids, and the dual-luciferase reporter assay was performed following the same protocol as described above. The results showed that the truncated fragment covering nucleotides -125 to -1 induced the highest luciferase activity, which was significantly higher than that of other secondary truncated fragments (Fig. 1H). Collectively, these findings confirm that the region spanning nucleotides -125 to -1 of the NUDT7 gene serves as its core promoter, which is crucial for mediating the transcriptional activation of NUDT7, and this core promoter region may be the key target through which PRRSV upregulates NUDT7 expression.

Based on the obtained results, we examined the potential transcription factor binding sites (TFBS) of the promoter with the JASPAR (http://jaspar.genereg.net/). ZNF354C-, KLF5-, SP1-, SP2-, KLF2-, KLF15-, TFAP2A- and ETS1-binding sites were held within the minimal NUDT7 key promoter region (Fig. 1I). Further analyses of all the predicted TFBS by RT-qPCR revealed that the ZNF352C, SP1 and ETS1 mRNA level were upregulated among other predicted transcription factors in PRRSV-infected PAM cells (Fig. 1J). Combined with the GenDoma database (https://ai.citexs.com), it was found that there was a correlation between ETS1 and NUDT7, so the regulation of transcription factor ETS1 was mainly studied. We also observed that the abundance of ETS1 protein was upregulated during PRRSV infection (Fig. 1K), which was consistent with NUDT7 endogenous expression tendency. To demonstrate whether ETS1 regulates the expression of NUDT7, a potential ETS1 binding site was identified in the promoter region of NUDT7 gene and deleted (Fig. 1L). Construct ETS1 expression plasmids, and co-transfected into PAM cells with the reporter plasmid or the reporter plasmid that deleted the ETS1 site. The results showed that overexpression of ETS1 could significantly upregulate the activity of NUDT7 promoter, and the deletion of binding sites led to the disappearance of the effect (Fig. 1M). We further showed that NUDT7 increased with the overexpression of ETS1 by western blot (Fig. 1N) and RT-qPCR (Fig. 1O), confirming that ETS1 positively regulated the activity of NUDT7 promoter and promoted its expression. Overall, these results suggest that NUDT7 expression is induced by PRRSV infection in an ETS1-dependent manner.

### PRRSV Nsp2 interacts with NUDT7

**Figure 2:**
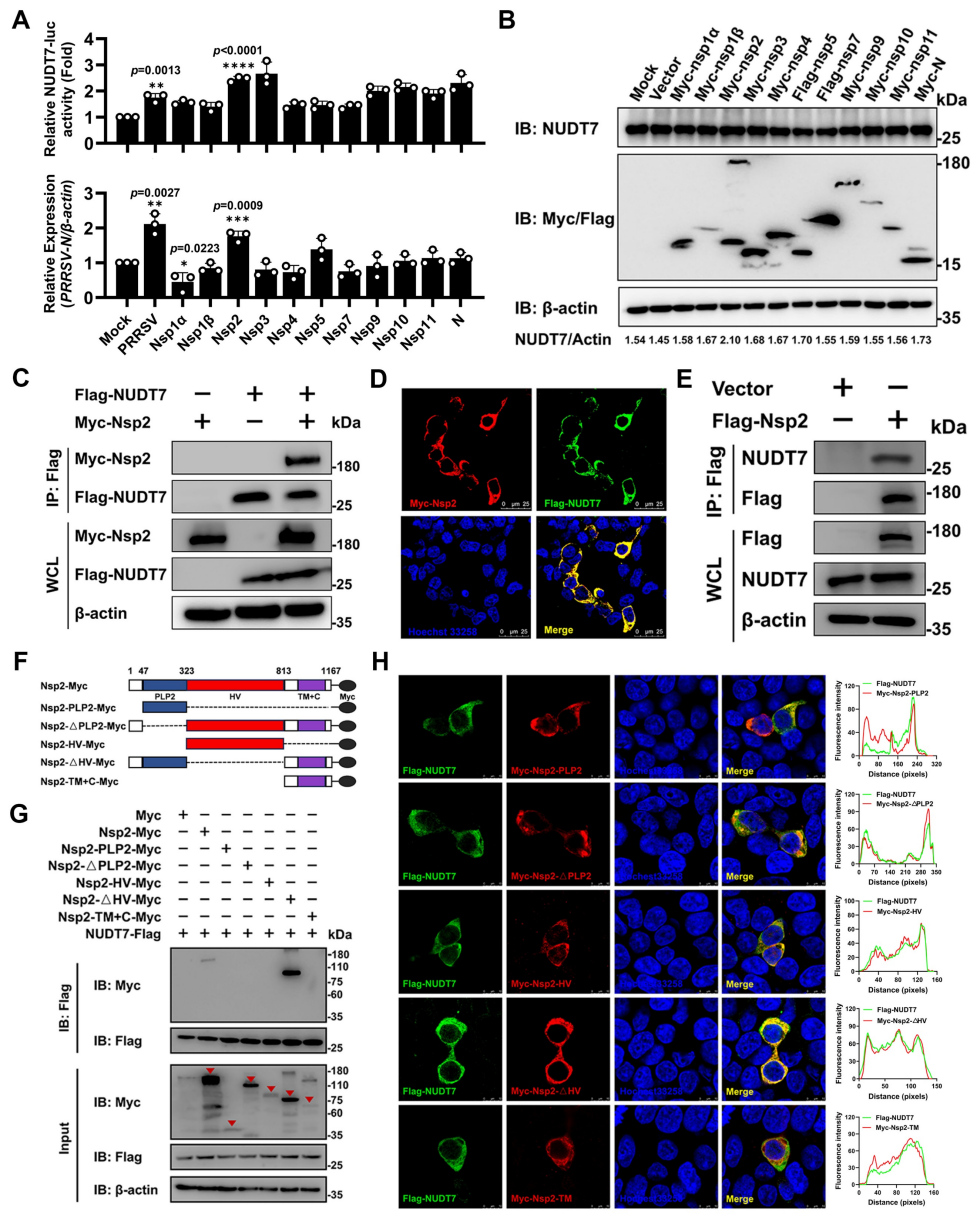
PRRSV Nsp2 interacts with NUDT7. (A) Expression vectors encoding PRRSV proteins were transfected into Marc-145 cells. NUDT7 levels were assessed using luciferase assays and RT-qPCR. (B) NUDT7 levels were assessed via western blotting 36 hours after transfection with expression vectors for PRRSV-encoded proteins. (C) An exogenous co-immunoprecipitation (Co-IP) assay was conducted in HEK293T cells transfected with Myc-Nsp2 and either Flag-NUDT7 or an empty vector. Following transfection, cell lysates underwent immunoprecipitation with a Flag antibody and subsequent immunoblotting with both Myc and Flag antibodies. (D) Marc-145 cells were transfected with MYC-NSP2 and FLAG-NUDT7, then subjected to immunofluorescence staining using anti-FLAG (green), anti-Myc (red), and Hoechst 33258 (blue). (E) Co-IP experiments showing the interaction of Flag-Nsp2 with endogenous NUDT7 in iPAM cells. (F) The schematic diagram of Myc-Nsp2 deletion mutants. (G) HEK293T cells were transfected for 36 hours with plasmids encoding Flag-NUDT7 and either Myc-Nsp2 or specified Myc-Nsp2 deletion mutants. Subsequent co-immunoprecipitation was performed using anti-Flag beads, followed by immunoblotting with anti-Myc and anti-Flag antibodies. Triangle labeled Myc-Nsp2 or specified Myc-Nsp2 deletion mutants. (H) Truncated Nsp2 and NUDT7 colocalization (Left). Pearson's correlation coefficient (Right) was used to express co-localization analysis, measured for individual cells using Image J. *p < 0.05; **p < 0.01; ***p < 0.001; ****p < 0.0001.

To screen PRRSV-encoded proteins responsible for NUDT7 upregulation, expression vectors encoding Myc/Flag-tagged PRRSV proteins were transfected into Marc-145 cells to detect NUDT7 promoter activity and mRNA expression. The results showed that PRRSV Nsp2 significantly promoted the expression of NUDT7 (Fig. 2A), and western blot also obtained similar results (Fig. 2B). Subsequently, we co-transfected the Myc-Nsp2 and Flag-NUDT7 plasmids in HEK293T cells and detected an interaction between exogenous NUDT7 and Nsp2 (Fig. 2C). Meanwhile, we observed the co-localization of Myc-Nsp2 and Flag-NUDT7 via confocal microscopy (Fig. 2D). Additionally, PRRSV Nsp2 could efficiently coimmunoprecipitate with the endogenous NUDT7 (Fig. 2E). These results collectively suggest that NUDT7 interacts with PRRSV Nsp2 protein.

PRRSV Nsp2 harbors a modular structure, including an N-terminal papain-like cysteine protease 2 (PLP2) domain with both cleavage and deubiquitination activities, a central hypervariable region (HV), a hydrophobic transmembrane region (TM), and a C-terminal tail (C)^28^. To identify which specific domain of Nsp2 is responsible for interacting with NUDT7, we constructed a panel of Myc-tagged PRRSV Nsp2 deletion mutants, which were designated as Nsp2-PLP2-Myc (PLP2 domain-only construct), Nsp2-ΔPLP2-Myc (PLP2 domain-deleted construct), Nsp2-HV-Myc (HV region-only construct), Nsp2-ΔHV-Myc (HV region-deleted construct), and Nsp2-TM+C-Myc (TM region and C-terminal tail fusion construct), respectively (Fig. 2F). Co-IP results showed that NUDT7 exhibited interaction with both the full-length PRRSV Nsp2 and its Nsp2-ΔHV, suggesting that the N-terminal and C-terminal regions of PRRSV Nsp2 were the primary interaction site with NUDT7 (Fig. 2G). Additionally, the subcellular localization of PRRSV NSP2 domain with the NUDT7 protein was investigated. While colocalize of Nsp2-ΔHV and NUDT7 was observed throughout the cytoplasm, the remaining domains of Nsp2 also showed partial co-localization with NUDT7 (Fig. 2H). In summary, these data indicate that PRRSV Nsp2 interacts with NUDT7.

### NUDT7 positively regulates PRRSV infection

**Figure 3:**
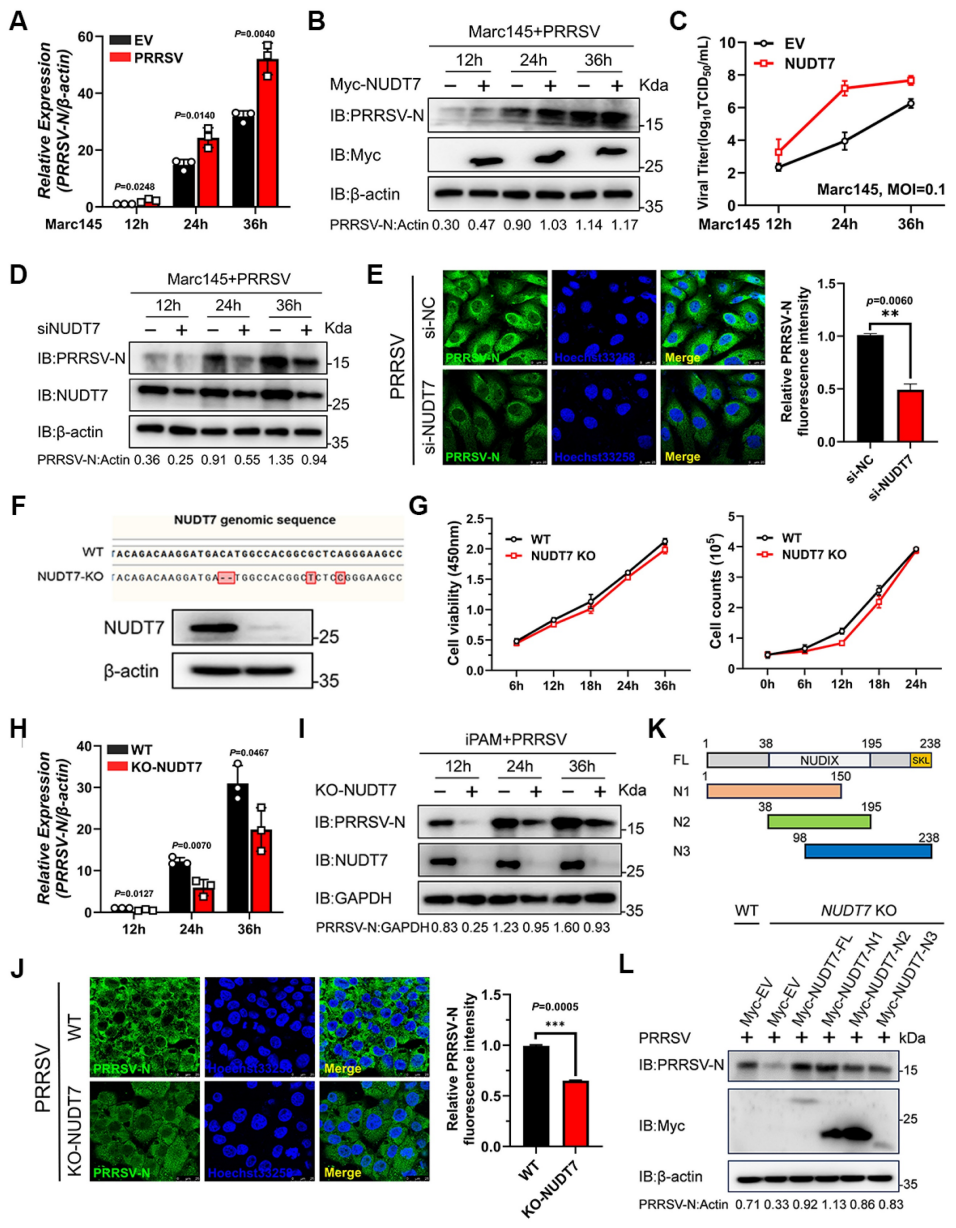
PRRSV infection was promoted by NUDT7. (A-B) Marc-145 cells were transfected with either a vector or Myc-NUDT7, then infected with PRRSV (MOI=1) for 12, 24, and 36 hours. PRRSV-N mRNA and protein levels were quantified using RT-qPCR and western blot analysis. Normalization was performed using β-actin as the internal control. (C) Marc-145 cells underwent identical treatment conditions, and the supernatant was collected for TCID_50_ analysis to assess viral titer. (D) Marc-145 cells were transfected with either siNC or siNUDT7 and subsequently infected with PRRSV at a multiplicity of infection (MOI) of 1. Western blot analysis was used to assess PEDV-N protein levels. (E) The N protein was visualized using an anti-N antibody (green), while the nuclei were counterstained blue. Fluorescence images were taken at 10x magnification (F-G) Sequence analysis was conducted on both wild-type (WT) and CRISPR-Cas9-mediated NUDT7 knockout (KO) iPAM cell lines. Additionally, the cell viability and growth kinetics of NUDT7 KO cells were compared with those of WT cells. (H-I) Wild-type (WT) and NUDT7 knockout (KO) cells were infected with PRRSV, followed by analysis of cell lysates using RT-qPCR and immunoblotting. (J) PRRSV-N levels were assessed via immunofluorescence in NUDT7 KO cells 24 hours post-PRRSV infection. (K) The schematic diagram of the truncated domain of NUDT7. (L) Wild-type (WT) or NUDT7 knockout (KO) cells were transfected with either full-length or truncated NUDT7 for 24 hours, followed by infection with PRRSV at a multiplicity of infection (MOI) of 1 for another 24 hours. Subsequent cell lysates were subjected to immunoblot analysis.

To clarify the regulatory effect of NUDT7 on PRRSV proliferation, Marc-145 cells were first transfected with NUDT7-overexpressing plasmids or empty vector (EV), as a negative control. At 24 h post-transfection, the cells were infected with PRRSV at a predetermined multiplicity of infection (MOI). Subsequently, cells were harvested at three different time points post-infection (hpi), namely 12, 24, and 36 hpi, for subsequent experimental detections including reverse transcription-quantitative PCR (RT-qPCR), western blotting, and 50% tissue culture infective dose (TCID₅₀) assays. The results showed that compared with EV-transfected control cells, ectopic overexpression of NUDT7 significantly upregulated the copy number of PRRSV genomic RNA (Fig. 3A), enhanced the expression level of PRRSV nucleocapsid (N) protein (Fig. 3B), and increased the viral titer (Fig. 3C). To further verify the role of NUDT7 in PRRSV replication, we designed three small interfering RNAs (siRNAs) specifically targeting NUDT7 to achieve NUDT7 knockdown. Western blotting results demonstrated that NUDT7 knockdown effectively suppressed PRRSV replication, as evidenced by the decreased expression level of PRRSV N protein (Fig. 3D). Additionally, indirect immunofluorescence assay (IFA) was performed to further confirm the effect of NUDT7 on PRRSV proliferation. Consistent with the aforementioned results, IFA data also verified that NUDT7 facilitates PRRSV infection (Fig. 3E).

To further verify this observation, we generated NUDT7 knockout (KO) iPAM cells using CRISPR/Cas9 gene-editing technology (Fig. 3F), and this disruption did not apparently affect the cell viability and growth kinetics (Fig. 3G). Then, PRRSV infection was performed on wild-type (WT) and NUDT7-knockout iPAM cell lines. Samples were collected at specified times to assess viral RNA, protein abundance, and fluorescence levels, respectively. The results showed that the absence of NUDT7 resulted in a significant decrease in PRRSV genome mRNA (Fig. 3H). Furthermore, N protein level was significantly decreased in NUDT7-KO iPAM cells compared to WT cells (Fig. 3I). Similarly, this result was also confirmed by IFA (Fig. 3J). The peroxisome targeting signal (PTS1) motif is the c-terminal tripeptide serine-lysine-leucine (-SKL), which is essential for cell metabolism by guiding and sorting proteins into the peroxisome. Here, multiple sequence alignments revealed that the PTS1 motif of NUDT7 is conserved across multiple species (Fig. S1A). To probe into the role of the PTS1 motif in PRRSV infection, we introduced serine-to-alanine and leucine-to-alanine substitutions into the WT NUDT7 plasmid (S235A+L237A) and found that NUDT7 mutant not impaired N expression (Fig. S1B-C), indicating that NUDT7 promotes PRRSV proliferation independent of the PTS1 motif. To identify which region of NUDT7 responsible for increasing viral replication, we generated three truncated mutants of NUDT7 (N1 aa 1-150, N2 aa 38-195, N3 aa 98-238) as shown in Fig 3K. Our results indicated that three truncations of NUDT7 protein could promote the replication of PRRSV in varying degrees (Fig. 3L). This result reveals that the region of NUDT7 responsible for PRRSV replication is not single and has functional redundancy. Collectively, the above results demonstrate that NUDT7 facilitates PRRSV replication.

### NUDT7 reprograms PRRSV-induced lipid metabolism

**Figure 4:**
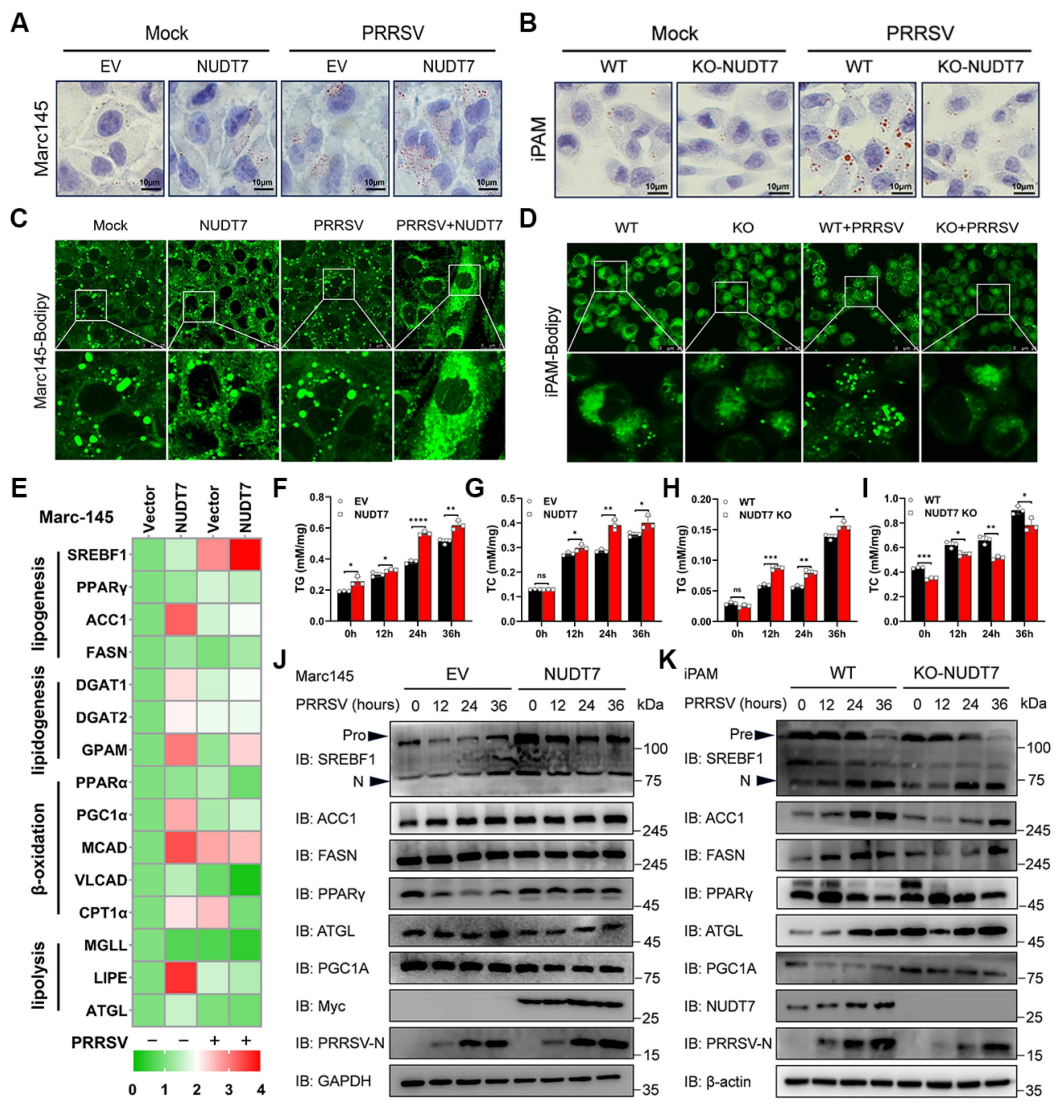
NUDT7 reprograms PRRSV-induced lipid metabolism. (A-B) Marc-145 cells, along with empty vector and Myc-NUDT7 transfected cells, were exposed to PRRSV for 24 hours. WT and NUDT7 KO cells were as described above (B). Oil Red O staining was used to analyze LDs. Scale bar: 10 μm. (C-D) LDs were detected by BODIPY staining. (E) RT-qPCR analysis of NUDT7-overexpressing Marc-145 cells revealed mRNA expression levels of several lipid metabolism-related factors. (F-I) PRRSV infected OE-NUDT7 Marc-145 cells or NUDT7-KO iPAM cells respectively. Cellular lipids were extracted, and TG and TC levels were quantified by biochemical kits. (J-K) PRRSV infected OE-NUDT7 Marc-145 cells or NUDT7-KO iPAM cells at specified time points, respectively. Analysis of lipid metabolism-related factors using Western blot.

Previous studies have shown that PRRSV infection enhances lipid synthesis and induces increased lipid droplets, which provides a platform for viral replication and assembly as well as a source of lipids energy^29^. Given that NUDT7 is involved in LD metabolism, we assume that PRRSV upregulates NUDT7 to utilize LD for viral replication. Oil red O staining in Marc-145 and iPAM showed that PRRSV infection increased the number of LDs (Fig. 4A-B). Meanwhile, NUDT7 promotes lipid droplet production, as demonstrated in NUDT7-overexpressing (OE) and -depleted (Knockout, KO) cells. BODIPY (Boron-Dipyrromethene), a dye that specifically labels neutral lipids and is commonly used to detect lipid droplets. Consistent with Oil Red O staining, NUDT7 overexpression significantly increased the number of lipid droplets (Fig. 4C), while knockout decreased the production of lipid droplets (Fig. 4D). Subsequently, we measured the lipid content in Marc-145 and iPAM cells in response to PRRSV infection and observed a significant increase in triglyceride (TG) and cholesterol (TC) in PRRSV-infected Marc-145 and iPAM cells (Fig. 4F-I). Moreover, TC levels in both NUDT7-OE (Fig. 4G) and NUDT7-KO (Fig. 4I) cells indicated that NUDT7 increased cellular cholesterol content. However, although TG content can be promoted in NUDT7-OE cells (Fig. 4F), TG content is also upregulated in NUDT7-KO cells (Fig. 4H), suggesting that the accumulation of NUDT7 metabolites may mediate multiple lipid metabolism regulatory pathways to varying degrees.

Lipid homeostasis is dependent on the balance between lipogenesis (storage), fatty acid β-oxidation (consumption) and lipolysis^30^. To identify the functional role of NUDT7 in lipid homeostasis, we thus examined the expression of genes involved in lipid metabolism. Among them, increased lipid synthesis gene expression (such as SREBF1 and PPARγ) and decreased lipolytic gene expression (such as MGLL and ATGL) demonstrated that NUDT7 reprograms PRRSV-induced lipid metabolism (Fig. 4E). Western blot analysis showed that overexpression of NUDT7 significantly promoted the expression of lipid synthesis genes SREBF1 and PPARγ (Fig. 4J). However, knockout of NUDT7 inhibited PRRSV-enhanced expression of SREBF1, ACC1 and FASN, and promoted the expression of lipolytic gene ATGL and fatty acid β-oxidized PGC1α (Fig. 4K). These results indicate that NUDT7 is involved in PRRSV-stimulated lipid synthesis.

### SREBF1 emerges as a critical regulator in NUDT7-dependent lipogenesis

**Figure 5:**
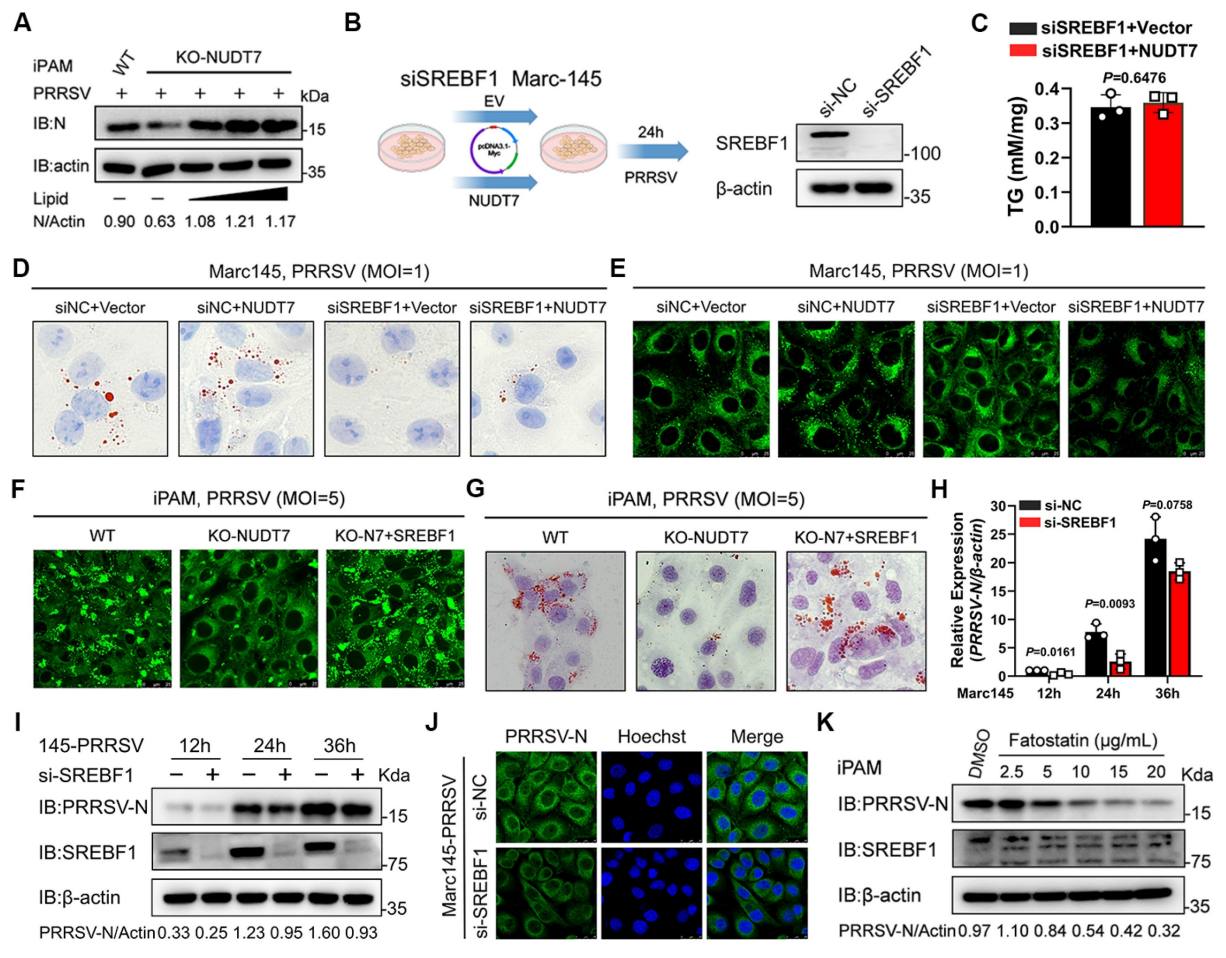
SREBF1 emerges as a critical regulator in NUDT7-dependent lipogenesis. (A) NUDT7 KO iPAM cells were treated with lipid (cholesterol and oleic acid) dose-dependent therapy and infected with PRRSV (MOI=1) for 24 h. The cell lysates were analyzed by immunoblotting. (B) The schematic diagram illustrates the experiment in which siSREBF1 cells were complemented with empty or NUDT7 and infected with PRRSV and the knockdown efficiency of SREBF1. (C) The triglyceride (TG) levels in siSREBF1 Marc-145 cells, transfected with either an empty vector or NUDT7 and subsequently infected with PRRSV, were analyzed. (D-E) Marc-145 was transfected with siRNA or siSREBF1 and complemented with NUDT7. Lipid droplets were stained with oil red O and Biodipy 24 hours post-PRRSV infection. Scale bar: 10 μm. (F-G) As previously described, PRRSV infection occurred after overexpression of SREBF1 in NUDT7 KO iPAM cells for 24 h. Cell lipid droplets are indicated with Oil Red O and Biodipy. Scale bar: 10 μm. (H-J) Marc-145 cells were transfected with siSREBF1 and subsequently infected with PRRSV at a designated time. PRRSV-N expression was analyzed using RT-qPCR, western blot, and immunofluorescence. (K) iPAM cells were exposed to PRRSV at an MOI of 1 and subsequently treated with either 10 µM Fatostatin or a DMSO vehicle control at a 1:1,000 dilution.

To determine the role of NUDT7-mediated lipid metabolism in PRRSV infection, we first examined whether lipid supplementation can salvage PRRSV replication in NUDT7-KO cells. RT-qPCR analysis showed that the number of PRRSV genomes in NUDT7-KO cells gradually increased with the increase of lipid concentration (Fig. S2A). Exogenous supplementation of lipid in NUDT7 knockout cells restored PRRSV virus replication, as indicated by immunoblotting analysis (Fig. 5A). Furthermore, immunofluorescence confirmed that lipid supplementation restored the damage of NUDT7 knockout on the PRRSV infection process (Fig. S2B-C). These results reveal that NUDT7 promotes PRRSV proliferation by reprogramming lipid metabolism.

Sterol regulatory element binding proteins (SREBFs) are a family of transcription factors that regulate lipid homeostasis, and cleavage of SREBFs release their N-terminal that could translocate to the nucleus and facilitate transcription^31^. Because we found that NUDT7 significantly regulates SREBF1 (Fig. 4J-K), we next examined whether NUDT7 reprogrammed lipid metabolism via SREBF1. For this purpose, the specific siRNAs against SREBF1 were designed and the interference efficiency was performed by western blot (Fig. 5B). Rescue experiment revealed that knocking down SREBF1 abolished the increased in triglyceride content caused by overexpressing NUDT7 (Fig. 5C). In addition, we discovered that SREBF1 knockdown significantly reduced LDs formation when compared to vehicle control. Meanwhile, SREBF1 knockdown partially abolished the number of LDs induced by NUDT7 overexpression (Fig. 5D-E). To further clarify the upstream/downstream relationship between lipid synthesis by NUDT7 and SREBF1, we also reintroduced SREBF1 or control vector into NUDT7-KO iPAM cells. Compared to empty vector control, SREBF1 reconstitution restored LDs production in NUDT7-KO iPAM cells, suggesting that decreased LDs production in NUDT7-KO cells was indeed caused by the reduced of SREBF1 (Fig. 5F-G). Since we confirmed that SREBF1 promotes lipid synthesis during PRRSV infection., we further investigated the role of SREBF1 in PRRSV infection. The results showed that knockdown of SREBF1 significantly downregulated the copy number of viral genome RNA (Fig. 5H), expression level of PRRSV N protein (Fig. 5I) and IFA further confirming that SREBF1 promotes PRRSV replication (Fig. 5J). Fatostain is a specific inhibitor of SREBF activation that inhibits adipogenesis. As expected, iPAM cell treatment with Fatostain significantly inhibited PRRSV replication (Fig. 5K). Collectively, taken together, these data suggest that NUDT7 promotes PRRSV replication by reprogramming lipid metabolism in a SREBF1-dependent manner.

### NUDT7 targets UBA52 promotes proteasomal degradation

**Figure 6:**
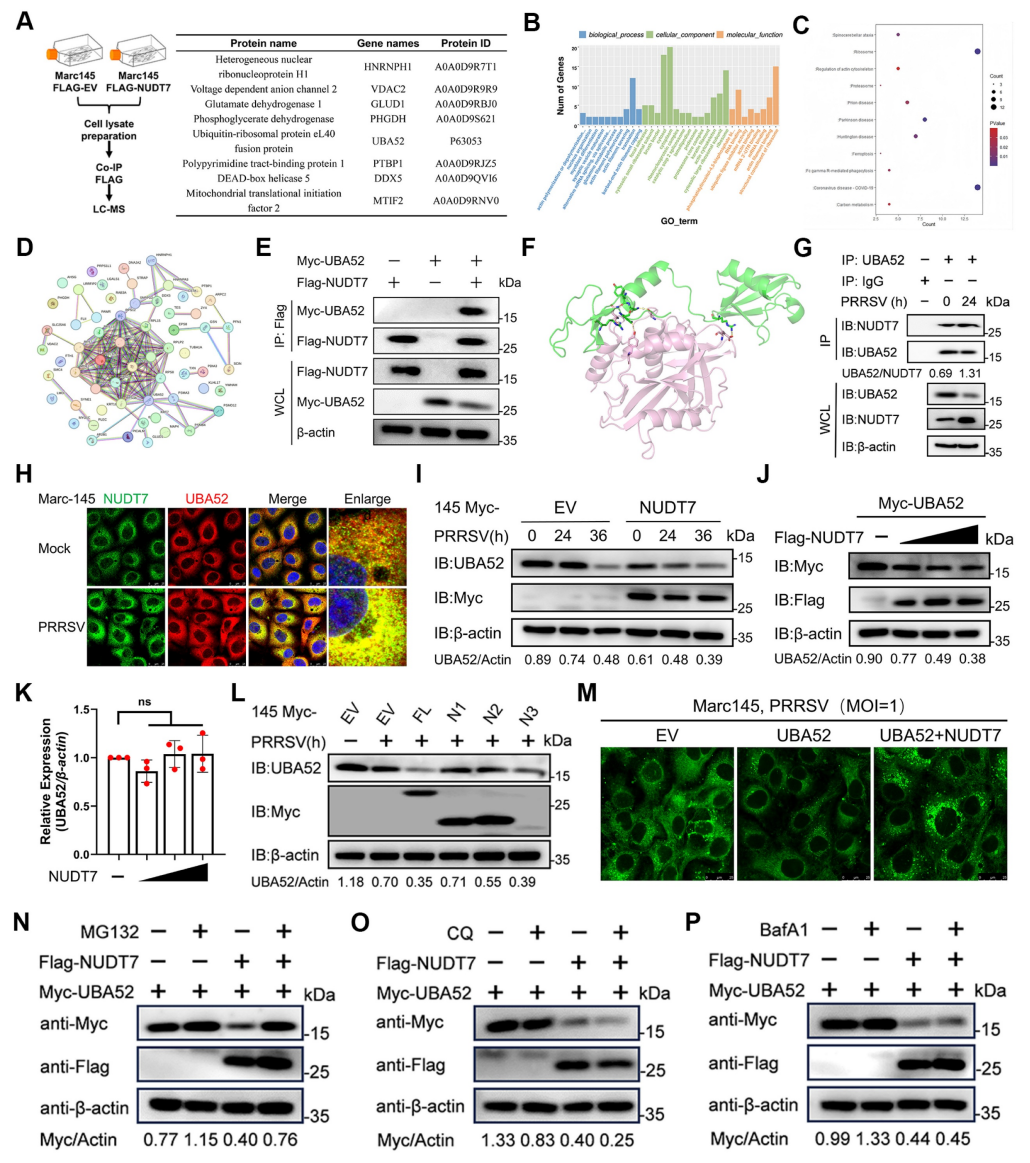
NUDT7 targets UBA52 to enhance proteasomal degradation. (A) Marc-145 cells were either transfected with an empty vector or FLAG-NUDT7 for 36 hours, followed by PRRSV infection (MOI=1) for 24 hours. Subsequently, the cells were lysed, and the supernatant from the lysate was analyzed using anti-Flag magnetic beads, with identification carried out through mass spectrometry. The proteins with high peaks that potentially interact with NUDT7 were enumerated. (B) GO enrichment analysis diagram. (C) KEGG pathway enrichment analysis results. (D) Interaction network graph illustrating protein-protein interactions (PPI). (E) Co-immunoprecipitation (Co-IP) assay was conducted in HEK293T cells, which were transfected with Myc-UBA52 and either Flag-NUDT7 or a control vector. After the transfection, cell lysates underwent immunoprecipitation using a Flag antibody followed by immunoblotting with Myc and Flag antibodies. (F) Molecular docking simulation of NUDT7 and UBA52 was performed utilizing ZDOCK software. (G) Co-immunoprecipitation (Co-IP) using anti-UBA52 and immunoblotting analysis were conducted on protein lysates from both control and iPAM infected with PRRSV (MOI of 5, 24 hours). (H) Infected Marc-145 cells with PRRSV (MOI of 1, 24 h) and control were labeled with the specified antibodies and examined through confocal microscopy. The UBA52 signal is shown in red, while NUDT7 is in green. The scale bars measure 20 μm. (I) Marc-145 cells were transfected with Myc-tagged NUDT7 for 24 hours and then subjected to PRRSV infection for 0, 12, and 24 hours. (J) An immunoblot was performed on protein extracts derived from Marc-145 cells that had been transfected with plasmids for Myc-UBA52 along with various amounts of Flag-NUDT7. (K) The UBA52 mRNA levels in Marc-145 cells transfected with Myc-UBA52 and differing quantities of Flag-NUDT7 were assessed through RT-qPCR analysis. Results are shown as relative fold changes, normalized against β-actin controls. (L) An analysis of protein extracts was carried out on Marc-145 cells transfected with plasmids encoding Myc-NUDT7 and its truncated forms. (M) The specified plasmid was transfected into Marc-145 for 24 hours. After being infected with PRRSV for an additional 24 hours, lipid droplets were visualized using Bodipy staining. Scale bar: 10 μm. (N-P) Immunoblot analysis on lysates from Marc-145 co-transfected for 24 hours with Flag-NUDT7 and Myc-UBA52 was performed, followed by treatment with various inhibitors 24 hours before PRRSV infection: MG-132 (10µM) (N), chloroquine (CQ, 20µM) (P), or NH4Cl (20 mM) (Q).

To further explore the molecular mechanism underlying NUDT7 regulation of SREBF1, co-immunoprecipitation (co-IP) assay was performed using transiently expressed Flag-tagged NUDT7 proteins as bait, along with anti-Flag magnetic beads and subsequently identified by mass spectrometry (MS) analysis. High-confidence candidate NUDT7 interacting proteins identified by mass spectrometry are indicated in Fig. 6A. Gene ontology (GO) analysis indicated that the interaction proteins were involved in multiple RNA splicing binding and ubiquitination pathways (Fig. 6B). Kyoto Encyclopedia of Genes and Genomes (KEGG) pathway enrichment analysis showed that the interacting proteins were significantly enriched for pathways involved in proteasome, ferroptosis, coronavirus disease-COVID-19 and carbon metabolism pathways (Fig. 6C). Protein-protein interaction network (PPI) shows the interactions between candidate proteins (Fig. 6D). Previous studies have shown that the SREBF1 transactivation domains (TAD) domain degrades by non-covalently bound to ubiquitin (UBI)^32^, and the ubiquitin A-52 residue ribosomal protein fusion product 1 (UBA52) with the UBI domain is highly enriched in mass spectrometry analysis. Therefore, we further analyzed the interaction between NUDT7 and UBA52. Co-IP results showed that NUDT7 could bind to UBA52 (Fig. 6E). In addition, protein interaction prediction for NUDT7 with UBA52 using molecular docking to further confirm the interaction between NUDT7 and UBA52 (Fig. 6F). Endogenous co-IP assay showed the interaction between NUDT7 and UBA52 enhanced after PRRSV infection in Marc-145 cells (Fig. 6G). Consistently, confocal microscopy analysis showed that NUDT7 had more colocalization with UBA52 in Marc-145 after PRRSV infection (Fig. 6H). Given that Nsp2 can interact with NUDT7 and enhance its expression, we further investigated whether Nsp2 affects the interaction between NUDT7 and UBA52. Co-IP experimental results show that exogenous overexpression of Nsp2 can enhance the interaction between UBA52 and NUDT7. In addition, overexpression of Nsp2 also exacerbated the degradation of UBA52, which might be attributed to the elevated expression level of NUDT7 (Fig. S3A). In summary, these results collectively suggest that NUDT7 interacts with UBA52.

Based on PRRSV-induced NUDT7 interaction with UBA52, we examined proteins levels of UBA52 by immunoblotting and found that UBA52 protein in WT NUDT7 cells was remarkedly reduced by PRRSV infection in a time-dependent manner (Fig. 6I) and in a dose-dependent manner (Fig. 6J). Decreased UBA52 protein was not due to lower gene transcription (Fig. 6K). We next attempted to identify the domain within the NUDT7 responsible for degrading UBA52. The results clearly indicate that amino acids 98-238 of NUDT7 are sufficient and necessary for degradation (Fig. 6L). In addition, BODIPY staining showed that UBA52 inhibited LDs formation after PRRSV infection. Reconstitution of UBA52-OE cells with WT NUDT7 significantly restored the number of LDs following PRRSV infection (Fig. 6M). Although the PTS1 motif does not regulate the proliferation of PRRSV, the 98-238aa of NUDT7 mainly degrades UBA52. Therefore, we explore whether NUDT7, which is located on the peroxisome, relies on the peroxisome to function. Therefore, we first examined the effect of NUDT7 on peroxisome generation. The results showed that the peroxisome marker protein PMP70 was significantly reduced during PRRSV infection, while overexpression of NUDT7 could reverse this phenomenon (Fig. S3B). Furthermore, when we treated the cells with 3-AT (a peroxisome inhibitor), the degradation effect of NUDT7 on UBA52 was significantly weakened, and Bodipy staining further verified this result (Fig. S3C-D). In conclusion, the degradation of UBA52 by NUDT7 depends on the function of peroxisomes.

To investigate the molecular mechanism by which NUDT7 mediates the degradation of UBA52, Marc-145 cells were co-transfected with plasmids encoding Flag-tagged NUDT7 (Flag-NUDT7) and Myc-tagged UBA52 (Myc-UBA52). After 24 hours of transfection, the cells were treated with either proteasome inhibitor (MG132) or autophagy inhibitors (bafilomycin A1, BafA1; chloroquine, CQ) for a predetermined period. Subsequent western blotting analysis was performed to detect the expression level of UBA52. Our results showed that the degradation of UBA52 induced by NUDT7 was completely abrogated by the proteasome inhibitor MG132. In contrast, the autophagy inhibitors BafA1 and CQ had no significant effect on NUDT7-mediated UBA52 degradation (Fig. 6N-P). These findings collectively suggest that NUDT7 interacts with UBA52 and promotes the proteasomal degradation of UBA52 during PRRSV infection.

### NUDT7 stabilizes SREBF1 by preventing UBA52-mediated SREBF1 polyubiquitination and degradation

**Figure 7:**
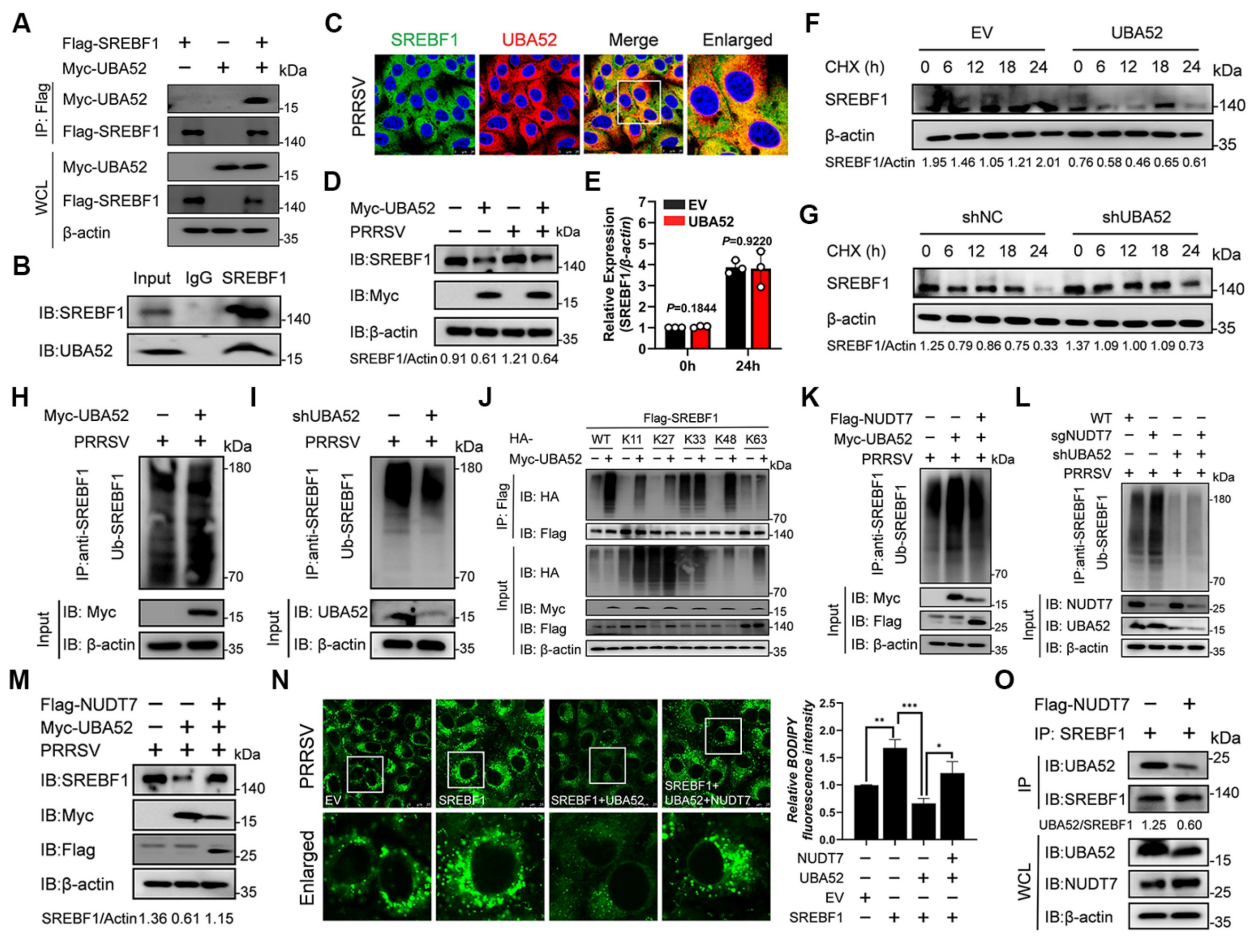
NUDT7 enhances the stability of SREBF1 by inhibiting UBA52-induced polyubiquitination and subsequent degradation of SREBF1. (A) Co-immunoprecipitation (Co-IP) assays were conducted in Marc-145 cells that were transfected with Myc-UBA52 and Flag-SREBF1 or control vectors. After 24 hours of transfection, cells were inoculated with PRRSV (MOI=5), and cell lysates were subjected to immunoprecipitation using Flag antibodies, followed by immunoblotting with Myc and Flag antibodies. (B) Examination of UBA52 and SREBF1 interactions through Co-IP in Marc-145 cells. (C) The distribution of SREBF1 and UBA52 in cells infected with PRRSV was assessed via immunofluorescence. Scale bar: 20 μm. (D) The levels of specific proteins in Marc-145 cells, either with UBA52 overexpression or subjected to PRRSV infection, were analyzed using Western blotting. (E) RT-qPCR was performed to evaluate the mRNA levels of SREBF1 in Marc-145 cells transfected with plasmids expressing Myc-UBA52. Results are shown as relative fold changes, normalized against β-actin controls. (F-G) Cells were treated with CHX (15 μM) for the specified duration, and the expression of SREBF1 was evaluated in the relevant groups. (H-I) The ubiquitination of SREBF1 was identified through Western blot analysis in both UBA52-overexpressing Marc-145 cells and UBA52 knockdown iPAM cells infected with PRRSV. (J-L) The examination of SREBF1 ubiquitination was conducted on Marc-145 cells that were transfected with specific constructs and subsequently infected with PRRSV. (M) Endogenous SREBF1 expression was detected via Western blotting after inoculating PRRSV 24 hours following the overexpression of the specified plasmids. (N) BODIPY staining showed the presence of lipid droplets in cells 24 hours post-PRRSV infection following the overexpression of the indicated plasmid in Marc-145 cells (left). Scale bar: 10 μm. The relative fluorescence intensity of BODIPY in the cells was quantified (right). (O) For 24 hours, Marc-145 cells were transfected with a Flag-tagged NUDT7 plasmid. Following cell lysis and centrifugation, the supernatant was incubated overnight with SREBF1 magnetic beads, and subsequent protein immunoblots were performed to detect proteins.

To elucidate whether UBA52 regulates lipid metabolism in PRRSV infection via SREBF1, we first examined whether UBA52 and SREBF1 interact with each other. Indeed, both exogenous and endogenous interactions of the two proteins were detected by Co-IP experiments (Fig. 7A-B). Findings of confocal microscopic analysis showed that UBA52 was co-localised with SREBF1 in the cytoplasm (Fig. C). These data suggest that there is an interaction between UBA52 and SREBF1 during PRRSV infection process. We next examined whether UBA52 regulates SREBF1-expression. Interestingly, we found that UBA52 overexpression reduced the protein level of SREBF1 (Fig. 7D), while it had no effect on the mRNA level of SREBF1 (Fig. 7E). Moreover, results from protein degradation assays demonstrated that the half-life of endogenously expressed sterol regulatory element-binding transcription factor 1 (SREBF1) was significantly reduced in UBA52-overexpressing (UBA52-OE) cells compared to that in empty vector-transfected control cells (Fig. 7F). Conversely, knockdown of UBA52 yielded the opposite effect, leading to a marked extension of the SREBF1 protein half-life (Fig. 7G). Notably, previous studies have reported that UBA52 modulates the stability of target proteins through a ubiquitin-proteasome system (UPS)-dependent pathway. Based on this, we further explored whether UBA52 promotes SREBF1 protein degradation via inducing its ubiquitination. To this end, Marc-145 cells were transfected with plasmids expressing Myc-tagged UBA52, and subsequent ubiquitination assays were performed. The results showed that the total ubiquitination level of SREBF1 was significantly elevated in UBA52-overexpressing cells, indicating that UBA52 can readily induce SREBF1 ubiquitination (Fig. 7H). Conversely, knockdown UBA52 in iPAM cells attenuated the total ubiquitination of SREBF1 (Fig. 7I). To further determine the specific type of polyubiquitination modification of SREBF1 mediated by UBA52, a series of ubiquitin (Ub) mutants with individual lysine (K) residues substituted (including K11, K27, K33, K48, and K63 mutants) were employed. These Ub mutants were co-transfected with relevant plasmids into Marc-145 cells to examine the effect of UBA52 on different types of SREBF1 polyubiquitination (Fig. 7J). The results revealed that UBA52 significantly enhanced K11-linked, K27-linked, and K48-linked polyubiquitination of SREBF1, whereas no obvious effect was observed on K33-linked or K63-linked polyubiquitination. Taken together, these findings demonstrate that NUDT7 facilitates the degradation of SREBF1 through a UBA52-mediated polyubiquitination pathway. To determine whether NUDT7 blocks UBA52-mediated SREBF1 degradation, immunoblotting and co-immunoprecipitation assays were performed. We found that overexpression of NUDT7 dramatically reduced UBA52-mediated polyubiquitination of SREBF1 (Fig. 7K). Additionally, a decrease in NUDT7 levels was observed to increase SREBF1 ubiquitination, and a decrease in UBA52 levels blocked this process (Fig. 7L). We also found that NUDT7 overexpression rescued the expression levels of SREBF1 initially downregulated by UBA52 overexpression (Fig. 7M). BODIPY staining further demonstrates that NUDT7 restores lipid droplet formation by inhibiting UBA52-mediated degradation of SREBF1 (Fig. 7N). Notably, in vitro binding assays showed that overexpression of NUDT7 reduced the formation of the UBA52-SREBF1 complex (Fig. 7O). Taken together, these results indicate that NUDT7 inhibits SREBF1 polyubiquitination and degradation by inhibiting UBA52 from binding to SREBF1.

### NUDT7 inhibits the IFN-I immune response upon viral infection

**Figure 8:**
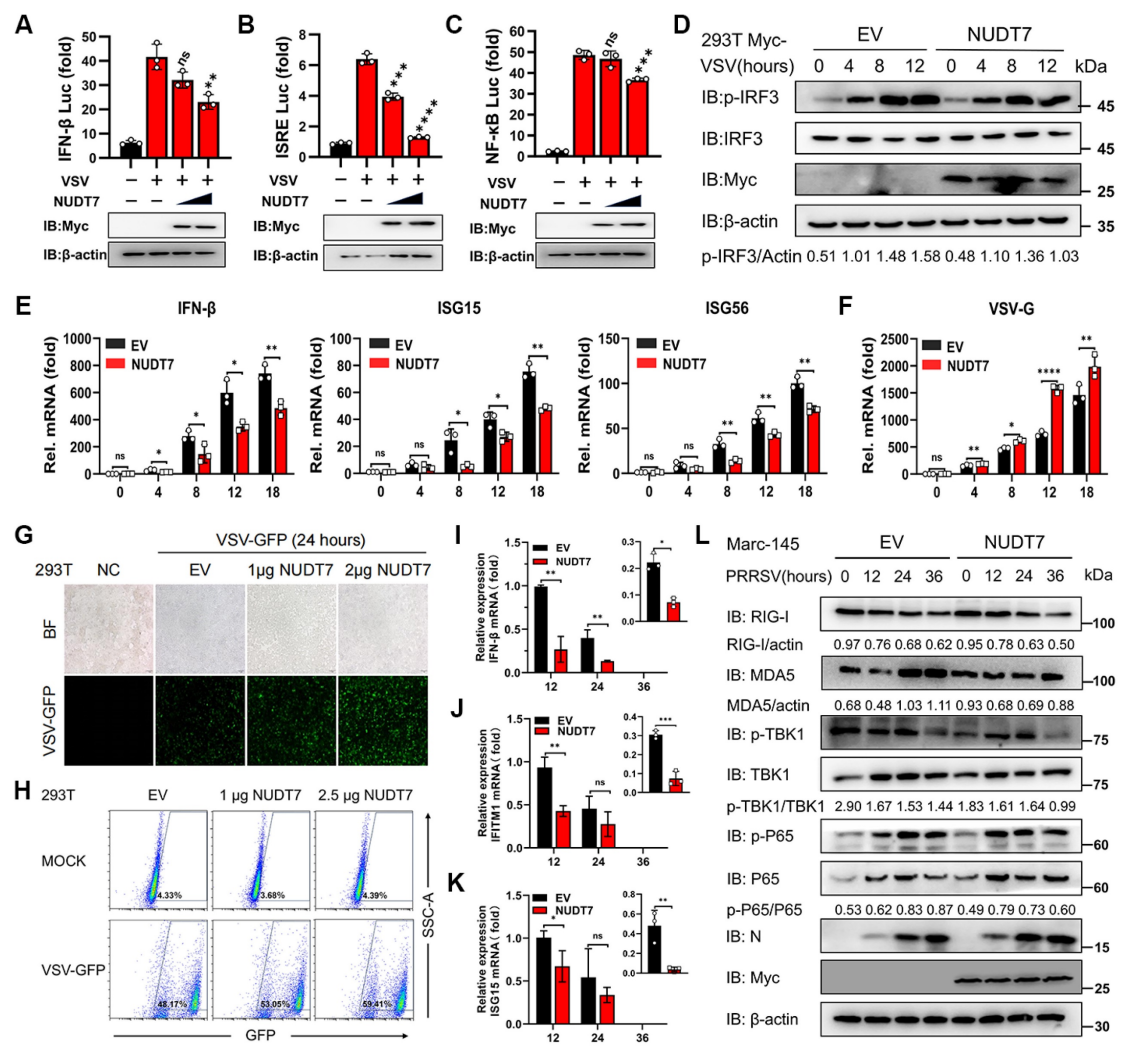
NUDT7 diminishes the IFN-I immune response during viral infection. (A-C) Luciferase reporter assays were conducted to evaluate the promoter activity of IFN-β, ISRE, and NF-kB in HEK293T cells that were transfected with escalating amounts of Myc-NUDT7 or an empty vector (EV) for 24 hours, subsequently treated with or without VSV for 18 hours. (D-F) Immunoblot analysis (D) of both total and phosphorylated IRF3, along with RT-qPCR assessments (E-F) of specified gene expression in HEK293T cells that underwent transfection with Myc-NUDT7 or EV for 24 hours, followed by VSV infection at defined time points. (G-H) Analysis using fluorescence microscopy (G) and flow cytometry (H) to investigate the replication of GFP-VSV in HEK293T cells transfected with either EV or varying amounts of Myc-NUDT7 at specified dosages for 24 hours, followed by treatment with or without GFP-VSV infection at designated time intervals. (I-L) Marc-145 cells were transfected with either EV or Myc-NUDT7 for 24 hours and subsequently infected with PRRSV. Cells were collected at 12-, 24-, and 36-hours post-infection (hpi) for RT-qPCR to assess mRNA expression levels (I-K) or for western blotting to determine protein expression levels (L).

Many recent studies have shown that type I interferon (IFN-I) production is affected by host cell metabolism, and NUDT7 can negatively regulate basic immunity and participate in maintaining redox homeostasis. Given the above findings, we next sought to evaluate the role of NUDT7 in regulating antiviral immune responses. To this end, 293T cells were transfected with NUDT7-overexpressing plasmids or empty vector (EV) as a control, followed by infection with vesicular stomatitis virus (VSV) (a classic model virus for investigating antiviral innate immunity). Luciferase reporter assay results showed that overexpression of NUDT7 significantly suppressed the activation of interferon-β (IFN-β) (Fig. 8A), IFN-stimulated response element (ISRE) (Fig. 8B), and nuclear factor-κB (NF-κB) responsive reporters induced by VSV infection. Consistently, western blotting and reverse transcription-quantitative PCR (RT-qPCR) analyses demonstrated that ectopic expression of NUDT7 markedly inhibited the phosphorylation of interferon regulatory factor 3 (IRF3) (a key downstream molecule in the IFN signaling pathway) and the transcription of antiviral genes, including IFN-β, ISG15 (interferon-stimulated gene 15), and ISG56 (interferon-stimulated gene 56), in VSV-infected 293T cells (Fig. 8D-E). Conversely, NUDT7 overexpression promoted VSV replication in these cells (Fig. 8F). Moreover, dose-dependent experiments showed that the viral burden was significantly increased in 293T cells transfected with increasing amounts of exogenous NUDT7 plasmids. This dose-dependent promotion of viral replication was consistent with the dose-dependent reduction in the expression levels of IFNs (Fig. 8G-H), further confirming that NUDT7 exerts a negative regulatory effect on antiviral immune responses.

We then proceeded to further investigate the role of NUDT7 in regulating IFN-β and ISG induction during PRRSV infection. To this end, Marc-145 were transfected with Myc-NUDT7 for 24 h and then infected with PRRSV for the indicated lengths of time. RT-qPCR results demonstrated that overexpression of NUDT7 significantly downregulated the mRNA expression levels of IFN-β, IFITM1, and ISG15 following PRRSV infection, compared with the vector control (Fig. 8I-K). In addition, western blot assay further shows that NUDT7 significantly inhibited the expression of the IFN-I signaling pathway proteins at 36 h after PRRSV infection (Fig. 8L). In general, these results suggest that NUDT7 during virus infection effectively inhibits IFN-I signaling and antiviral immunity. To investigate the interaction between NUDT7 and innate immune signaling molecules, we co-transfected Myc-NUDT7 with various immune proteins tagged with Flag into HEK293T cells. Regrettably, our findings indicate that NUDT7 does not interact with RIG-IN, MAVS, TRAF3, TBK1, IKKi, and IRF3 (Fig. S4A). We next hypothesize whether NUDT7-UBA52-SREBF1 axis reprogrammed lipid metabolism promotes viral replication by inhibiting IFN-I. RT-qPCR analysis indicated that the PRRSV transcripts N gradually increased with an increased concentration of cholesterol and oleic acid (Fig. S4B), suggesting that lipid synthesis could promote viral replication. But we found that exogenous lipid supplementation simultaneously also promoted the expression of IFN-I and ISGs (Fig. S4C-E). Therefore, we hypothesize that the proviral effect of lipid synthesis mediated by the NUDT7/UBA52/SREBF1 axis is not dependent on the inhibition of IFN-I. Next, we evaluated the antiviral activity of NUDT7 against PEDV (porcine epidemic diarrhea virus) and PCV2 (porcine circovirus). Marc-145 cells and 3D4/21 were separately transfected with NUDT7 for 24 h, followed by inoculation with PEDV or PCV2 for an additional 24 h. RT-qPCR results revealed that both PEDV and PCV2 infections upregulated NUDT7 mRNA expression to varying extents. Moreover, NUDT7 overexpression significantly enhanced the replication of both viruses, as evidenced at the RNA level (Fig. S4F). These findings suggest that the evolutionarily conserved NUDT7 may exhibit broad-spectrum antiviral properties.

## Discussion

**Figure 9:**
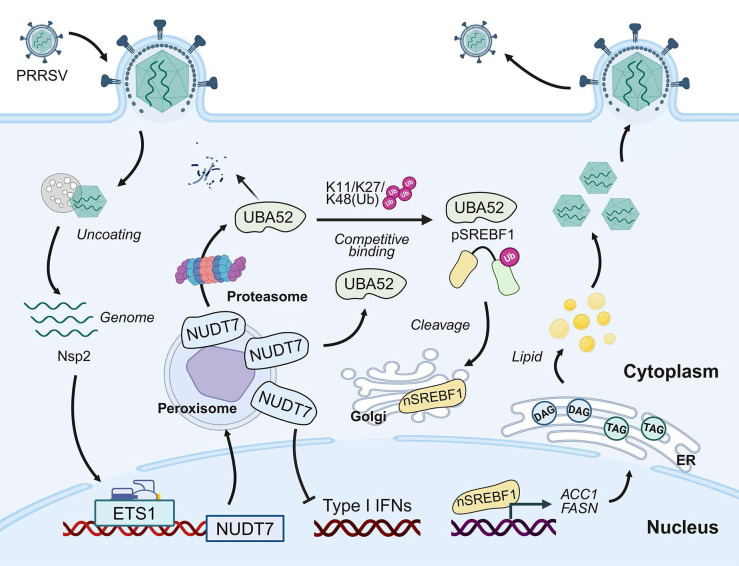
NUDT7 is a key host factor for the upregulation of PRRSV through ETS1 and directly interacts with viral Nsp2. NUDT7 enhances lipid droplet formation by blocking UBA52-mediated K11/K27/K48 polyubiquitination and proteasomal degradation of SREBF1. In addition, NUDT7 antagonizes type I interferon signaling and promotes immune evasion.

Understanding the mechanism of virus-host interaction is of great significance for understanding the pathogenesis of virus infection and for the development of antiviral drug. In this study, we identify NUDT7 as a critical host factor for PRRSV infection and pathogenesis. We established that LD accumulates significantly after PRRSV infection but is inhibited at NUDT7 deletion, indicating lipid metabolism in viral infection reprogramming cells. PRRSV infection enhances cellular lipid synthesis through the NUDT7/UBA52/SREBF1 axis and promotes PRRSV replication. In addition, NUDT7 can antagonize the production of IFN-I and assist the virus in achieving immune escape (Fig. 9).

The NUDT (Nudix hydrolase) family is a superfamily of phosphohydrolases that are widely present from archaea to humans. In mammals, NUDT exhibits a wide range of functions and is characterized by different substrate specificity and intracellular localization^33^. NUDT1, also known as MTH1, can selectively hydrolyze oxidized nucleotides (such as 8-oxo-dGTP, 8-oxo-dATP), preventing them from blending into DNA and causing mutations. It is the "guardian" of genomic stability^34^. NUDT5, as an ADP-ribose pyrophosphatase, participates in regulating purine metabolism. It directly binds to PPAT (purine synthesis rate-limiting enzyme) through a non-catalytic scaffold effect, inhibiting de noblo purine synthesis^35^. NUDT21 is a component of the precursor mRNA cleavage and polyadenylation complex. It regulates variable polyadenylation (APA) through lactation modification, influencing gene expression and tumor drug resistance^36^. NUDT family proteins, as the "regulatory hub" of cellular metabolism, participate in key processes such as genomic protection, RNA processing, and energy metabolism by hydrolyzing various nucleotide derivatives. Meanwhile, transcriptome revealed that NUDT7 was significantly regulated by the PRRSV, indicating that NUDT7 plays a crucial role in viral replication. NUDT7, a peroxisomal acyl-CoA hydrolase, plays a crucial role in hydrolyzing specific nucleic acid metabolites to uphold intracellular metabolic equilibrium. Prior research has predominantly examined NUDT7's function as a CoA bisphosphatase in preserving cellular redox balance, detoxifying harmful metabolites, and its involvement in the inflammatory response. Notably, perfluorinated compounds such as PFOA/PFOS have been shown to reduce NUDT7 transcription, subsequently impeding Ace-CoA hydrolase activity^37^. NUDT7 concurrently influences cholesterol excretion and fatty acid synthesis through the CoA-bile acid and PPARγ-DNL pathways. A promoter regions analysis of NUDT7 revealed a direct repeat 1 (DR1- TGACCTGTGACCT) could bind to PPARα/RXR, and RAR/RXR dimers. Furthermore, RAR and RXR were considered to be binding sites of NUDT7 transcription factors in GeneHancer database^38^,^39^. Therefore, the result implied that the transcription of NUDT7 gene might be regulated by PPARα, RAR and RXR. In this study, the function of NUDT7 in virus-host interaction was investigated for the first time, and our data showed that NUDT7 expression was significantly induced by PRRSV, and this induction process was dependent on the transcription factor ETS1. More importantly, the viral protein Nsp2 has been shown to have a direct interaction with NUDT7, which may not only stabilize the NUDT7 protein, but also regulate the function of NUDT7 through its deubiquitinase activity. Of particular note is that multiple truncated bodies of NUDT7 can promote viral replication to varying degrees, suggesting redundancy in its functional domains, which illustrates the importance of the protein's function from an evolutionary perspective.

Cellular lipids, essential biomolecules, play a critical role in both cellular function and viral life cycles^40^. PRRSV infection upregulates the expression of pivotal proteins that govern lipid metabolism and the quantity of LDs within cells. Particularly, SREBF1 serves as a crucial transcription factor in lipid biosynthesis, orchestrating lipid synthesis through the upregulation of key enzymes including Acetyl-CoA Carboxylase 1 (ACC1), ATP Citrate Lyase (ACLY), Stearoyl-CoA Desaturase 1 (SCD1), and Fatty Acid Synthase (FASN)^41^,^42^. Recent research has revealed that PRRSV Nsp4 degrades the lipolytic enzyme ATGL and triggers the ROS-SREBFs axis to manipulate host lipid metabolism, leading to the accumulation of triglycerides and lipid droplets that facilitate its replication^29^,^43^. Conversely, the host employs let-7f-5p to suppress SREBF2, along with transcription factors YY1 and STEAP3, to reconfigure fatty acid synthesis and lipid droplet formation as defense mechanisms against lipid droplet-associated diseases^44^-^46^. Consequently, the modulation of lipid metabolism emerges as a compelling strategy for the prevention and management of PRRSV. Our data suggest that inhibition of NUDT7 expression leads to a reduction in lipid droplet numbers during PRRSV infection, and triglyceride and cholesterol levels are positively correlated with NUDT7 expression. In addition, up-regulation of NUDT7 promoted changes in lipid metabolism-related protein levels, while down-regulation of SREBF1 inhibited lipid droplet production induced by NUDT7 overexpression. These results suggest that SREBF1 is a key factor in NUDT7-mediated lipid reprogramming during PRRSV infection. Therefore, targeting the NUDT7-SREBF1 axis may be a potential therapeutic strategy for antivirals.

We then investigated the mechanism by which NUDT7 regulates SREBF1 expression. Luciferase reporter gene detection showed that NUDT7 did not regulate SREBF1 promoter activity (Fig. S4E). Mass spectrometry results showed that NUDT7 interacting proteins are involved in multiple biological pathways, including RNA-binding splicing and substrate ubiquitination. Pyrimidine bundle binding protein 1 (PTBP1) has been reported to bind to SREBF1 mRNA precursor, thereby regulating its mRNA stability and alternative splicing^47^. Thus, siRNAs designed for PTBP1 have been used to explore the transcriptional mechanisms of SREBF1 (Fig. S4F). However, NUDT7 did not promote SREBF1 mRNA production via PTBP1 (Fig. S4G-H). Therefore, how NUDT7 promotes SREBF1 mRNA levels is a key question to be solved in the current study. Based on the existing studies, it is speculated that its core mechanism may involve miRNA regulation, mRNA transport and metabolite/signal pathway mediation. Ubiquitination modification of SREBF1 is a key mechanism for protein stability and functional regulation. Many E3 ubiquitin ligases (e.g. RNF20, TRIM21, ARMC5) and deubiquitin enzymes (e.g. USP11) participate in its dynamic regulation, affecting lipid metabolism, cell proliferation and tumorigenesis^48^-^51^. As an E3 ubiquitination ligase, UBA52 is a newly identified NUDT7 interacting factor. UBA52 significantly inhibits lipid accumulation by inhibiting ACC1 and FASN expression through K11/K27/K48-related ubiquitination mediated SREBF1 degradation. Through a series of proteomic experiments, we found that NUDT7 inhibits ubiquitination and degradation of SREBF1 through competitive binding with UBA52. NUDT7 and SREBF1 compete with UBA52 for binding. NUDT7 reduced the formation of UBA52-SREBF1 complex and ultimately increased the expression level of SREBF1. Taken together, our data clearly showed that NUDT7 stabilizes SREBF1 by preventing its degradation mediated by UBA52.

IFN-I is essential for host defense against invading viruses. Past studies have indicated that NUDT7 regulates basal immunity in plants^27^. Our investigation revealed that upregulation of NUDT7 inhibited the transcription of IFN-β and ISGs, indicating assists PRRSV in achieving immune escape. In conclusion, our study highlights the dual function of NUDT7 in enhancing PRRSV replication through the integration of antiviral innate immune responses and lipid metabolism. This expands our knowledge of the interplay between immune signaling cascades and glycolipid metabolic pathways, emphasizing the significance of immune metabolism in diseases associated with viral infections.

## Supplementary Material

Supplementary figures and tables.
