# Supplementary material for: NUDT7 Modulates the UBA52-SREBF1 Signaling Axis to Promote PRRSV Replication via Lipid Synthesis
Source: Int J Biol Sci. 2026 Jan 14;22(3):1590–610. doi: 10.7150/ijbs.127844 (PMC12839166; doi:10.7150/ijbs.127844)
Supplement: Supplementary file 1 — Supplementary figures and tables. [file ijbsv22p1590s1.pdf]

Supplementary Figure

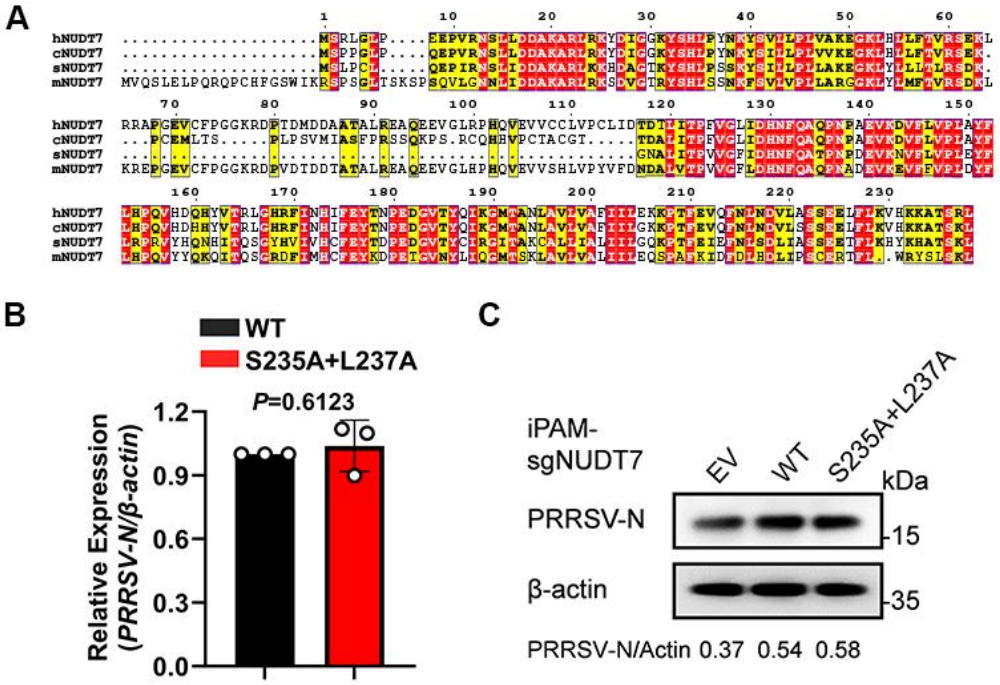

S1 Fig. The NUDT7 PTS1 motif does not affect PRRSV replication. (A) amino-acid sequence alignment of NUDT7 from various species. hNUDT7, the NUDT7 of human; mNUDT7, the NUDT7 of mouse; sNUDT7, the NUDT7 of Sus scrofa; cNUDT7, the NUDT7 of green monkey. This alignment was created using ClustalW. (B-C) WT and PTS1mutant plasmids were transfected into NUDT7 knockout cells and PRRSV was inoculated for 24 h. PRRSV-N gene was detected by RT-qPCR and Western blot. Actin as control. Data presented are means ±SD from triplicate experiments. *, ** and ***, respectively indicate $P < 0.05$, $P < 0.01$ and $P < 0.001$ (two-tailed Student's t-test).

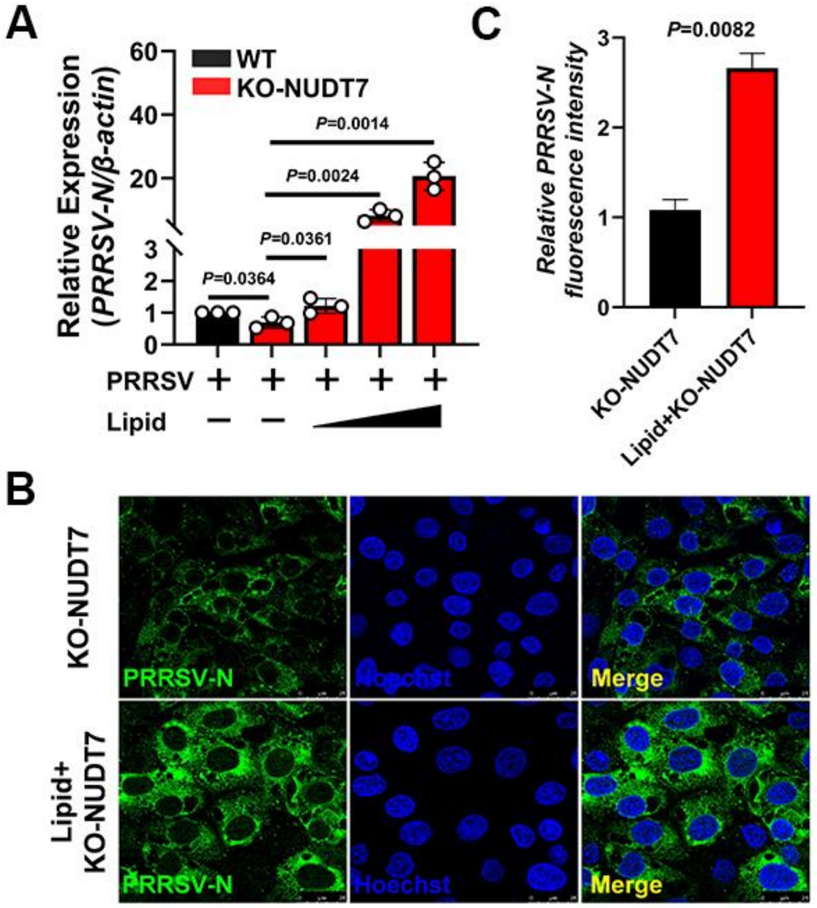

S2Fig. NUDT7 promotes PRRSV by reprogramming lipid metabolism. (A-C) NUDT7 KO iPAM cells were treated with lipid (cholesterol and oleic acid) dose-dependent therapy and infected with PRRSV (MOI=1) for 24 h. PRRSV-N expression was detected by qRT-PCR (A) and indirect immunofluorescence (B-C). All the data are processed by Student's t-test and shown as means ± SEM of three independent experiments (*, $p < 0.05$; **, $p < 0.01$; and ***, $p < 0.001$).

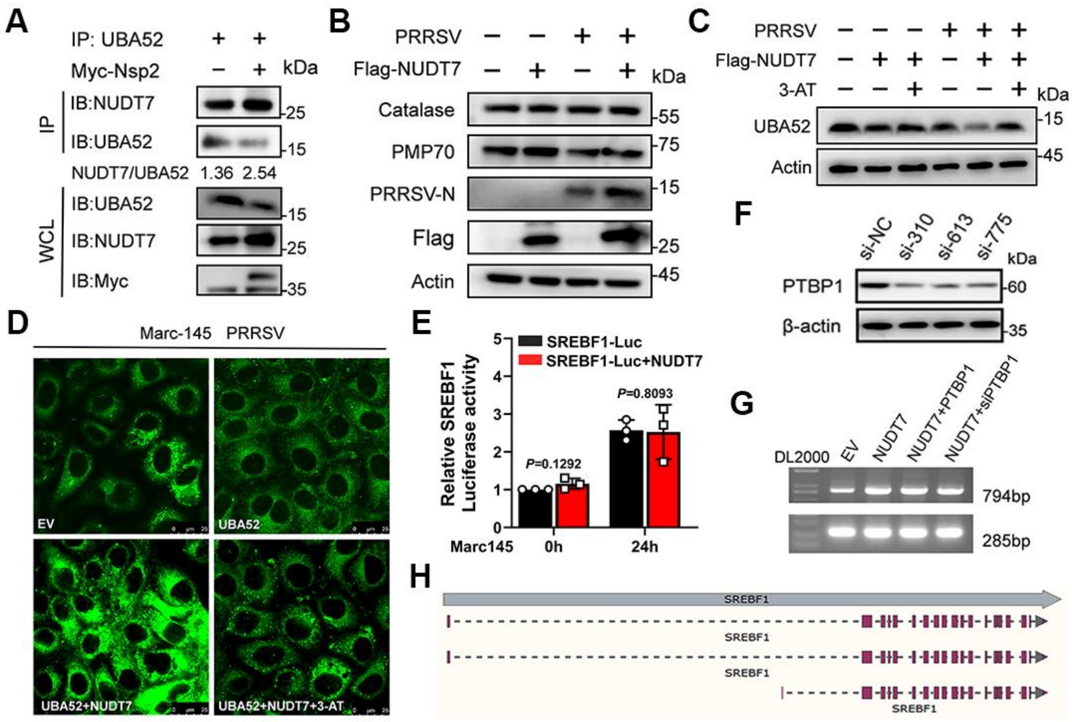

S3.Fig. (A) Myc-Nsp2 transfected HEK293T cells for 24 h, and the cells were collected. Co-IP experiment (anti-UBA52) was conducted and Western blotting analysis was performed using endogenous protein lysate. (B) Marc-145 cells were transfected with Flag-NUDT7 for 24 h, and infected or uninfected with PRRSV(MOI=1) for 24 h. Cell lysates were collected to detect the target protein. (C) Marc-145 cells were pretreated with 3-AT for 24 h, transfected with Flag-NUDT7 plasmid for 24 h to infect uninfected PRRSV, and the cell lysates were collected to detect the endogenous target protein. (D) Marc-145 cells were transfected with the corresponding plasmid and pretreated with 3-AT. The number of lipid droplets in the cells was detected by Bodipy staining. (E) Marc-145 cells were co-transfected with pcDNA-NUDT7, pGL3-SREBF1 and pRL-TK for 24 h, and then the luciferase activity and renin luciferase activity were measured at 0 or 24 h after PRRSV infection. (F) Western blot verification of PTBP1 siRNA knockdown efficiency. (G-H)

RT-PCR was used to assess splicing in Marc-145 co-expressing NUDT7 and PTBP1, to analyse the ability of NUDT7 to regulate splicing of SREBF1 pre-mRNA. The 794 bp product represents normal splicing. All the data are processed by Student’s t-test and shown as means ± SEM of three independent experiments (*, $p < 0.05$; **, $p < 0.01$; and ***, $p < 0.001$).

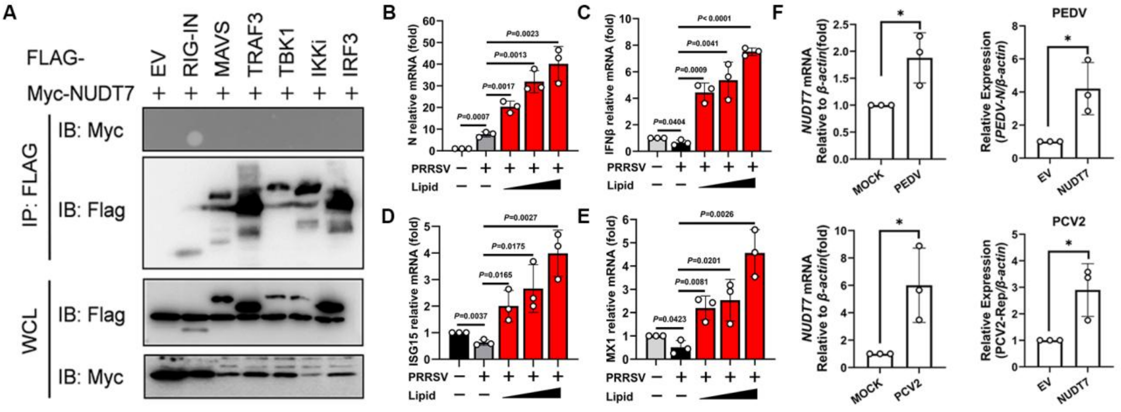

S4.Fig. (A). Myc-NUDT7 was co-transfected with various natural immune signaling molecules of Flag into HEK293T cells respectively. Collect the cell lysate, and use the FLAG magnetic bead immunoprecipitation complex to detect the expression of the corresponding protein by protein immunoblotting. (B-E) iPAM cells were treated with lipid (cholesterol and oleic acid) dose-dependent therapy and infected with PRRSV (MOI=1) for 24 h. PRRSV-N (B), IFN-β (C), ISG15 (D) and MX1 (E) were detected by RT-qPCR. (F) PEDV and PCV2 infected or uninfected Marc-145 and 3D4/21 cells respectively, and Myc-NUDT7 transfected the corresponding cells and inoculated the virus 24 h later respectively. The expression of the corresponding target gene was detected by qRT-PCR. All the data are processed by Student’s t-test and shown as means ± SEM of three independent experiments (*, $p < 0.05$; **, $p < 0.01$; and ***, p

< 0.001).

Table S1 Primer sequences for qRT-PCR in this study.

**Primer sequences of qRT-PCR in *sus scrofa***	
**Names**	**Sequences (5'→3')**
susNUDT7-F	GGAGCCCATCAGAAATAGT
susNUDT7-R	TAGCCAGCAGTGGTAAAAG
susβ-actin-F	GAATCCTGCGGCATCCACGA
susβ-actin-R	CTCGTCGTACTCCTGCTTGCT
susKLF2-F	CGTCCTTCTCCACTTTCGC
susKLF2-R	TGTTGAGGTCGTCGTCGGT
susKLF15-F	CCTATGCTGCTACGATTGC
susKLF15-R	CGGGGTTACTCCTCCTATT
susSP1-F	TACAGGGGTCTGATGCTC
susSP1-R	CTGTCCACCTTGAACTAACT
susSP2-F	GGAGGTGGCAATGTGA
susSP2-R	TCCTGGCTTTCTTGTTAGT
susTFAP2A-F	GCCATCCCCATCAACAAG
susTFAP2A-R	TGGAGCTGAGGAGCGAGA
susZNF354C-F	TCTTAGGCAAATGGTGGC
susZNF354C-R	ATGTTGGGTATCCGTTCT
susETS1-F	CAGTCGTCCTTCAACAGC
susETS1-R	CGGTCACGCACATAGTC
susKLF15-F	AGCATCGGGGCTAACGG
susKLF15-R	CTCGGGGAGGCAGAAAT
susIFN-β-F	GTTGCCTGGGACTCCTCAAT
susIFN-β-R	TGACGGTTTCATTCCAGCCA
susMx1-F	CTGCATCGACCTCATCGACT
susMx1-R	GCATCTTGTCACAATTCCGCT
susISG15-F	CTGGTGAGGAACGACAAGGGT
susISG15-R	AGCCAGAACTGGTCAGCTTGC

Primer sequences of qRT-PCR in Chlorocebus sabaeus	
**Names**	**Sequences (5’→3’)**
chlSREBF1-F	GACCTGCTGGATCTGCGAG
chlSREBF1-R	AGCCTTCTCTACAGGGAGCC
chlPPARγ-F	CCACTCCCACTCCTTTGAC
chlPPARγ-R	CCATCGGATCTGTTCTTGT
chlACC1-F	GGCAGCTCTGGAGGTGTATG
chlACC1-R	AGCGTAGGGATGTTCCCTCT
chlFASN-F	CAGGCGCTCAAGAAGGTGAT
chlFASN-R	ATTGTACTCGGCGGAAGACG
chlDGAT1-F	GACTACTCACGCATCATCG
chlDGAT1-R	AACTCCCGGTCTCCAAAC
chlDGAT2-F	CCAAGGTGGAAAAGCAGC
chlDGAT2-R	CAGCCAAGTGAAGTAGAGC
chlGPAM-F	CACAGCCGTTTTCCTTA
chlGPAM-R	AGCAGCATCATTGGGTC
chlPPARα-F	GCTGCTATCATTTGCTGTG
chlPPARα-R	TGAAGAAGTTTTGGGAAGA
chlPGC1A-F	CACAACACGGACAGAACT
chlPGC1A-R	TATAACGGTAGGTAATGAAAC
chlMCAD-F	ACAGGGGTTCAGACTGCTAT
chlMCAD-R	CTTCTTCTTTTGTTGCTCAT
chlVLCAD-F	CCCCTGTGGAAAATACTA
chlVLCAD-R	TGGCAAAGACTGTGAAGA
chlCPT1A-F	ATTACGTGAGCGACTGGT
chlCPT1A-R	GCTGCCTGAATGTGAGTT
chlMGLL-F	CCAAGAGCCAGGACAAGA
chlMGLL-R	GGAAGACGGAGTTGGTGA
chlLIPE-F	AAGCCTTTGAGATGCCACTG
chlLIPE-R	GATGAGCCTGACTAGGACGG
chlPNPLA2-F	CCCAGAGGACGAGGATGAGG
chlPNPLA2-R	GCAGGTGCTCCAGGATGTGA
chlNUDT7-F	ATTCCATCCTTTTGCCATT
chlNUDT7-R	AGGGTCACGCTTACCTCC
chlβ-actin-F	CTTAGTTGCGTTACACCCTTTC
chlβ-actin-R	TGTCACCTTCACCGTTCCA
chlUBA52-F	GACCAGCAGCGTCTGATAT
chlUBA52-R	CGGAGGGAAGGCTCAATA

Primer sequences of qRT-PCR in Homo sapiens	
**Names**	**Sequences (5’→3’)**
hIFNβ-F	GCTTGGATTCCTACAAAGAAGCA
hIFNβ-R	ATAGATGGTCAATGCGGCGTC
hISG15-F	CGCAGATCACCCAGAAGATCG
hISG15-R	TTCGTCGCATTTGTCCACCA
hISG56-F	TTGATGACGATGAAATGCCTGA
hISG56-R	CAGGTCACCAGACTCCTCAC
hGAPDH-F	GGAGCGAGATCCCTCCAAAAT
hGAPDH-R	GGCTGTTGTCATACTTCTCATGG

Primer sequences of qRT-PCR for virus detection	
**Names**	**Sequences (5’→3’)**
PRRSV-N-F	CAGTCAATCAGCTGTGCCAAA
PRRSV-N-R	ATCTGACAGGGCACAAGTTCCA
VSV-G-F	CAAGTCAAAATGCCCAAGAGTCACA
VSV-G-R	TTTCCTTGCATTGTTCTACAGATGG
PEDV-F	AGATCGCCAGTTTAGCACCA
PEDV-R	GGCAAACCCACATCATCGT

Table S2 The sequences of siRNAs/sgRNA/shRNA used in this study.

The sequences of siRNAs used in this study.		
**Names**	**sense (5’-3’)**	**antisense (5’-3’)**
si-chlNUDT7-1	GAAACAGUUUGCUAGAUGA	UCAUCUAGCAAACUGUUUC
si-chlNUDT7-2	AGGUUCAAUUUAAUCUUAA	UUAAGAUUAAAUUGAACCU
si-chlNUDT7-3	AUUCUCACUUGCCAUAUAA	AUUCUCACUUGCCAUAUAA
si-SREBF1	GCACUGAGGCUAAGCUGAATT	UUCAGCUUAGCCUCAGUGCTT
si-chlPTBP1-310	CCAACACCAUGGUGAACUATT	UAGUUCACCAUGGUGUUGGTT
si-chlPTBP1-613	GCACAGUGUUGAAGAUCAUTT	AUGAUCUUCAACACUGUGCTT
si-chlPTBP1-775	GCCUCAACGUCAAGUACAATT	UUGUACUUGACGUUGAGGCTT

The sequences of sgRNAs used in this study.	
sg-NUDT7-1	TCCGGTCTGACAAGCTAAGA
sg-NUDT7-2	CACCTACCTTGTCAGACCGG

The sequences of shRNAs used in this study.	
shUBA52	GCCCAGAGACACCAAAGAGTT
